# Extracellular vesicle–mediated GALNT1/RPRD1A glycosylation axis drives immune escape and peritoneal metastasis in gastric cancer

**DOI:** 10.1186/s13046-026-03759-7

**Published:** 2026-07-02

**Authors:** Guohua Jin, Jianguang Zhang, Qingying He, Shiyu Chang, Zheyu Dong, Yang Shi

**Affiliations:** 1 Department of Gastroenterology, The First Hospital of Jilin University, No. 1 Xinmin Street, Chaoyang District, Changchun, Jilin Province 130021 China; 2 The First Clinical Medical College of Jilin University, Changchun, 130021 China

**Keywords:** Gastric cancer, Peritoneal metastasis, Immune escape, Extracellular vesicles, GALNT1, RPRD1A, Glycosylation, CD8⁺ T cells, Lipid nanoparticles

## Abstract

**Background:**

Peritoneal metastasis represents one of the most lethal clinical manifestations of gastric cancer (GC) and is closely associated with immune evasion. Extracellular vesicles (EVs) have emerged as critical mediators of tumor–immune communication; however, the molecular mechanisms by which EV-associated glycosylation regulates immune escape and metastatic dissemination remain largely undefined.

**Methods:**

EVs derived from highly metastatic GC cells were isolated and functionally characterized in vitro and in humanized immune system mouse models. Their clinical relevance was evaluated using paired human GC specimens. Underlying mechanisms were investigated through genetic manipulation, lectin-based glycosylation assays, and immune cell co-culture experiments.

**Results:**

We identified GALNT1 as an EV-enriched glycosyltransferase that promotes GC cell invasion and suppresses CD8^+^ T-cell activation. Mechanistically, GALNT1-dependent O-GalNAc glycosylation of RPRD1A was associated with increased RPRD1A protein abundance without altering its mRNA expression. Increased RPRD1A acted as a key downstream effector driving immune escape and peritoneal metastasis. Clinically, GALNT1 and RPRD1A were significantly upregulated in GC tissues, positively correlated with each other, associated with reduced T-cell infiltration, and predictive of poor prognosis. In humanized mouse models, EV-mediated transfer of GALNT1 accelerated peritoneal dissemination and impaired antitumor immunity, whereas disruption of the GALNT1/RPRD1A axis restored T-cell infiltration and suppressed metastatic progression.

**Conclusions:**

These findings identify an EV-driven GALNT1/RPRD1A glycosylation axis as a previously unrecognized mechanism underlying immune escape and peritoneal metastasis in GC and support this pathway as a potential therapeutic target for advanced disease.

**Graphical Abstract:**

**Figure:**
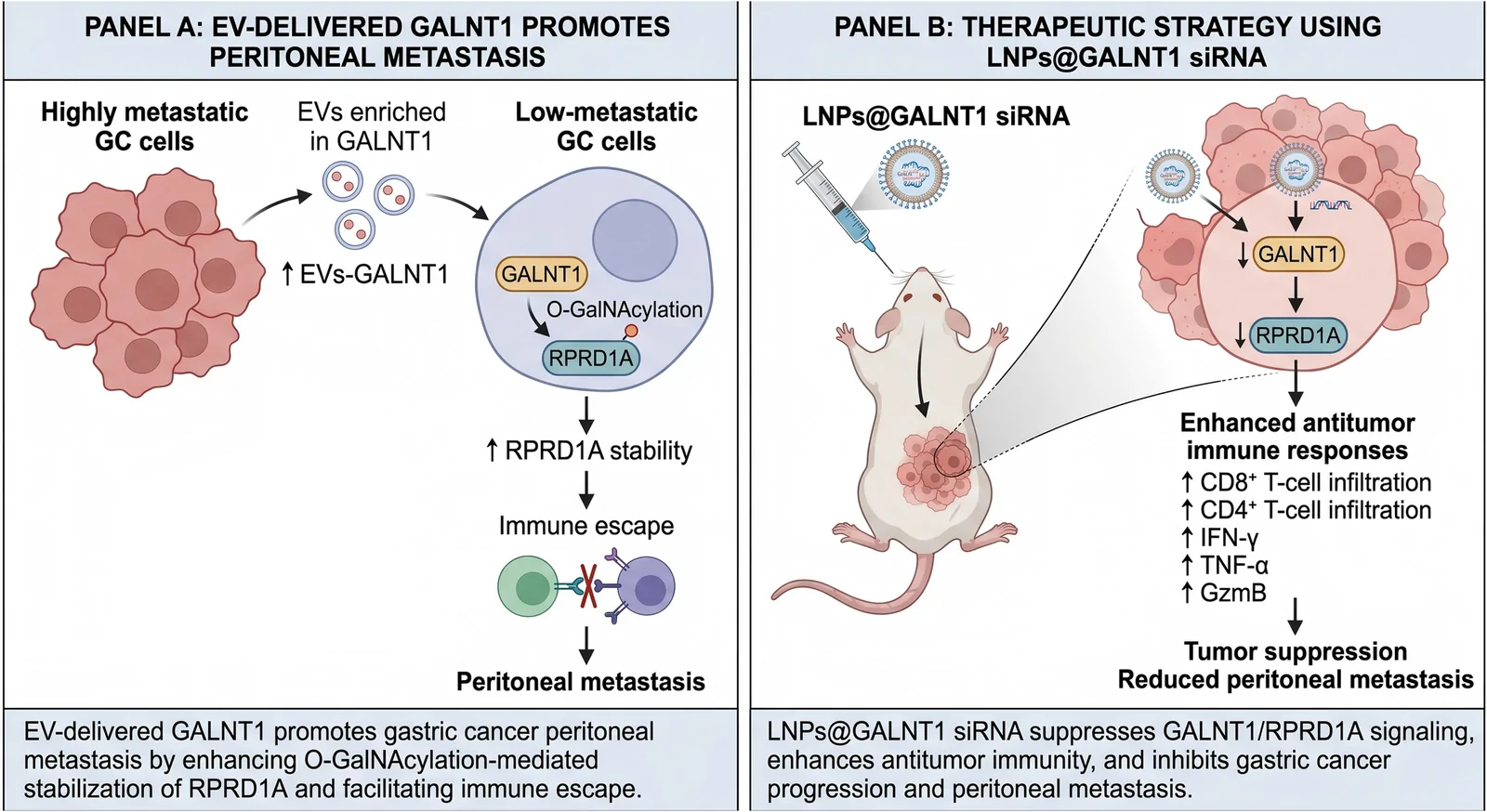

**Supplementary Information:**

The online version contains supplementary material available at https://doi.org/10.1186/s13046-026-03759-7.

## Background

Peritoneal metastasis is one of the most frequent and lethal metastatic patterns in gastric cancer (GC) [1]. Its development is driven by multiple factors, including the intrinsic properties of tumor cells, host immune responses, and genetic alterations [2]. Furthermore, growing evidence shows that the peritoneal microenvironment, composed of stromal cells, extracellular matrix components, and cytokines, supports the survival and outgrowth of disseminated GC cells [3]. Peritoneal metastasis is strongly associated with poor prognosis in patients with advanced GC [4, 5]. Although effector immune cells, particularly T cells and natural killer cells, are essential for recognizing and eliminating malignant cells, tumor cells can evade immune surveillance by upregulating immune checkpoint molecules and establishing an immunosuppressive microenvironment [6]. These adaptations facilitate immune escape, sustained tumor growth, and ultimately peritoneal metastatic spread [7].

GC remains a highly prevalent and life-threatening malignancy in China. Epidemiological studies have shown that its incidence has increased rapidly in recent years [8]. Notably, nearly 60% of patients are diagnosed at an advanced stage with extensive local invasion or distant metastasis, which severely limits treatment options and leads to poor clinical outcomes [9, 10]. Therefore, clarifying the molecular mechanisms underlying GC progression and metastasis is essential for the development of effective targeted therapies. In this context, tumor-derived extracellular vesicles (EVs) have attracted considerable attention because of their pivotal roles in tumor progression. As key mediators of intercellular communication, EVs transfer bioactive cargos, including proteins and nucleic acids, to recipient cells within the tumor microenvironment (TME), thereby reshaping the tumor milieu and promoting malignant phenotypes. Increasing evidence indicates that EVs contribute to tumor growth, invasion, metastasis, and immune escape [11, 12].

EVs are nanosized membrane vesicles released by various cell types, including cancer cells, and serve as important mediators of intercellular communication within the TME [13, 14]. In tumors, EVs can alter the TME by carrying cancer cell-specific proteins, nucleic acids, and bioactive molecules, thereby promoting tumor proliferation, invasion, and metastasis [15–17]. In GC, accumulating evidence indicates that GC-derived EVs actively remodel the TME, thereby enhancing the invasive and metastatic potential of tumor cells [18, 19].

Immune escape is a critical mechanism driving GC metastasis [20]. Within the TME, the activation and effector functions of immune cells, particularly T cells, are frequently suppressed, enabling tumor cells to evade immune surveillance and disseminate more efficiently [21]. EVs are important regulators of this process [22]. Tumor cells can impair immune cell function and promote tumor progression by delivering immunosuppressive factors through EVs or through direct interactions with immune cells [23, 24].

However, the EV-associated molecules that mediate immune escape in GC, particularly during peritoneal metastasis, remain poorly defined. Glycosylation, one of the most prevalent post-translational protein modifications, profoundly influences protein stability, subcellular localization, and molecular interactions, and plays a central role in remodeling the tumor immune microenvironment [25, 26]. Among these, N-acetylgalactosaminyltransferase 1 (GALNT1), the initiating enzyme of O-GalNAc glycosylation, a major form of mucin-type O-glycosylation, catalyzes glycosylation at specific sites on proteins [27]. Previous studies have shown that GALNT1 is aberrantly expressed in multiple tumors and is associated with malignant behaviors such as proliferation, invasion, and metastasis [28, 29]. Nevertheless, whether GALNT1 regulates immune cell infiltration and remodels the tumor immune microenvironment to promote immune escape remains largely unknown. RPRD1A (regulation of nuclear pre-mRNA domain-containing protein 1 A) is a protein involved in nuclear pre-mRNA processing and regulation of RNA polymerase II-associated transcriptional processes [30]. Although RPRD1A has been investigated in several non-neoplastic biological contexts [31, 32], its specific role in tumor development and metastasis has not been fully elucidated. Recent studies have begun to explore its potential functions in cancer, particularly in the regulation of the TME. Therefore, we further examined GALNT1-dependent O-GalNAc modification of RPRD1A in GC cell-derived EVs and investigated its role in immune escape and peritoneal metastasis in GC.

Lipid nanoparticles (LNPs) are a well-established platform for nucleic acid delivery and have shown considerable promise in mRNA vaccines, gene therapy, and targeted cancer treatment because of their biocompatibility, high nucleic acid loading capacity, and favorable in vivo stability and tunability [33–35]. By adjusting particle size, surface charge, and chemical composition, LNPs can be engineered to preferentially target specific cell types. Moreover, surface functionalization with tumor-targeting ligands, such as EpCAM antibodies, enables selective recognition of tumor-associated membrane antigens, thereby improving intratumoral accumulation and reducing off-target toxicity [36, 37]. Compared with conventional chemotherapy, LNP-based systems provide more efficient local gene silencing with fewer systemic adverse effects [38]. In this study, we developed EpCAM antibody-modified lipid nanoparticles loaded with GALNT1 siRNA (LNPs@GALNT1 siRNA) to target GALNT1, a key molecule associated with immune escape and metastasis in GC. This strategy was designed to silence GALNT1 expression in tumor cells, disrupt the GALNT1/RPRD1A signaling axis, inhibit invasion and metastasis, and restore T cell-mediated antitumor immunity. However, whether this approach can effectively suppress peritoneal metastasis and remodel the immune microenvironment in GC remains to be fully determined.

However, the key EV-associated molecules derived from highly metastatic GC cells that drive immune escape during peritoneal metastasis, as well as the underlying mechanisms, remain poorly understood. On the basis of the evidence described above, we hypothesized that highly metastatic GC cells may transfer specific glycosyltransferases, particularly GALNT1, through EVs to modify critical proteins in recipient cells, including tumor cells and immune cells, thereby coordinately promoting immune escape and peritoneal dissemination. To test this hypothesis, we sought to identify the key functional molecules enriched in EVs from highly metastatic GC cells, define their downstream signaling pathways in tumor cells, determine their effects on immune cells, and evaluate a nanotherapeutic strategy targeting this pathway.

Accordingly, the present study systematically investigated how EVs derived from highly metastatic GC cells contribute to immune evasion and peritoneal metastasis. The results showed that GALNT1 promoted tumor invasion and suppressed T cell function by increasing RPRD1A protein abundance through GALNT1-dependent O-GalNAc glycosylation. We further evaluated the therapeutic potential of EpCAM-modified lipid nanoparticles loaded with GALNT1 siRNA (LNPs@GALNT1 siRNA) in vitro and in humanized immune system (HIS) mouse models, with particular attention to tumor suppression, immune restoration, and biosafety. Collectively, these findings identify the GALNT1/RPRD1A axis as an important regulator of immune microenvironment remodeling and metastatic progression in GC, and support the feasibility of nanoparticle-based targeting of this pathway as a potential therapeutic strategy for advanced disease.

## Methods

### Bioinformatics analysis methods

RNA-sequencing data were downloaded from The Cancer Genome Atlas (TCGA) database (https://cancergenome.nih.gov/), including raw count data from 375 GC tissues and 32 non-tumor tissues. Corresponding clinicopathological information was also collected. Data were organized and extracted using Perl scripts. Differential expression of GalNAc transferases from GALNT1 to GALNT20 was extracted, and differential expression of GALNT genes was selected based on |log_2_FC|>1 and *p*.value < 0.05 criteria. Heatmaps and volcano plots were generated using R software.

### Correlation analysis of core genes with clinical pathological information and immune cell infiltration

The CIBERSORT algorithm (R script v1.03) was used to estimate the relative proportions of 22 immune cell types in GC samples. The “corrplot” package in R determined the relationships between different immune cells. The relationship between core genes and different immune cells was examined using the CIBERSORT R script obtained from the CIBERSORT website. Furthermore, based on CIBERSORT results and the TIMER database (https://cistrome.shinyapps.io/timer/), the correlation between core gene expression and GC immune cell infiltration was analyzed [39–43].

### Clinical sample collection

Tumor tissues and paired adjacent normal tissues were collected from 68 patients with GC who underwent surgical treatment at our hospital. All patients received radical gastrectomy and were pathologically diagnosed with GC after surgery. All surgical specimens were independently reviewed by experienced pathologists. Tumor-free distal tissues were cut into small pieces, snap-frozen in liquid nitrogen, and stored at − 80 °C until further use. The study was approved by the Ethics Committee of the First Hospital of Jilin University (2025 − 492). All patients or their guardians signed written informed consent and strictly adhered to the Helsinki Declaration. See Table S1 for the clinical characteristics of the patients.

Based on this cohort, samples from 10 patients with highly metastatic GC were further selected to analyze the association between patient-derived EVs and immune cell infiltration. Tumor-derived EVs were isolated from these patients, and GALNT1 expression in EVs was examined by western blot(WB). Corresponding formalin-fixed, paraffin-embedded tumor tissues were collected for immunohistochemical staining of CD8 and CD4. Under the same magnification, multiple representative fields were randomly selected, and the numbers of CD8^+^ and CD4^+^ T cells were quantified per unit area. Spearman’s rank correlation analysis was then performed to assess the associations between GALNT1 expression in patient-derived EVs and the infiltration levels of CD8^+^ and CD4^+^ T cells in matched tumor tissues. A two-sided *p <* 0.05 was considered statistically significant.

### In vitro cell culture

Human normal gastric mucosal cell line GES-1 (CBP60512), human GC cell lines AGS (CBP60476), SNU-520 (CBP60506), FU97 (CBP60478), and SNU-719 (CBP60511), as well as the highly metastatic human GC cell line MKN74 (CBP60490), were purchased from Nanjing KeyGen Biotech. Co., Ltd. (Nanjing, China), and the human GC peritoneal highly metastatic cell line GC9811-P (HTX2509) was purchased from Shenzhen Haodi Huatuo Biotech Co., Ltd. (Shenzhen, China). GC9811-P and AGS cells were cultured in RPMI 1640 medium (Art.No: 11875093) containing 10% fetal bovine serum (FBS; Art.No: 10099141 C) and 1% penicillin-streptomycin (Art.No: 10378016); the remaining cells were cultured in DMEM medium (Art.No: 11965092) containing 10% FBS (Art.No: 10099141 C) and 1% penicillin-streptomycin (Art.No: 10378016). All cells were maintained in a humidified incubator with 5% CO2 at 37 °C. The mentioned reagents were all purchased from ThermoFisher (USA).

### Separation of EVs

EVs were isolated from tumor tissues of 10 patients with highly metastatic GC and from GC9811-P cells using the Ultracentrifugation Ribo™ EVs Isolation Reagent (Art. No. C10130-1; RiboBio). Tumor-derived EVs were isolated as previously described with minor modifications [44]. Firstly, the tissues were washed with pre-chilled 0.1X DPBS and dried with sterile gauze. Then, the tissues were minced, digested in DMEM with 0.5 mg/mL type IV collagenase and 0.1 mg/mL DNase I, and incubated at 37 °C, 500 rpm for 1 h. The cell suspension was filtered through a 70 μm cell strainer for subsequent collection of EVs.

For cell-derived EV isolation, GC9811-P cells were cultured for 72 h in medium supplemented with EV-depleted FBS (Art. No. EVs-FBS-50 A-1; System Biosciences). The culture supernatant was collected and centrifuged at 2,000 × g for 30 min at 4 °C to remove floating cells, cell debris, and contaminating proteins. The clarified supernatant was transferred to a fresh tube on ice, mixed thoroughly with one-third volume of Ribo™ EVs Isolation Reagent, and incubated at 4 °C overnight. On the following day, 2 mL of the mixture was transferred into a centrifuge tube and centrifuged at 1,500 × g for 30 min at 4 °C. The supernatant was discarded and the pellet was retained. This step was repeated three to four times until all samples had been processed. The final EV pellet was resuspended in 1× phosphate-buffered saline (PBS) for subsequent experiments [45].

### Identification of GC9811-P-EVs by Transmission Electron Microscopy (TEM), Dynamic Light Scattering (DLS), and WB identification of EVs

For TEM analysis, 30 µL of EV suspension was placed onto a copper grid and allowed to stand for 1 min. Excess liquid was gently removed with filter paper. The grid was then incubated with 30 µL of phosphotungstic acid solution (pH 6.8) for 5 min at room temperature (RT), air-dried, and examined using a TEM (Talos F200X S/TEM; Thermo Fisher Scientific).

For particle size measurement, EV samples were diluted in 0.15 M NaCl at a ratio of 1:50 and mixed thoroughly before analysis using a Zetasizer Nano ZS90 instrument (Malvern, UK) with an excitation wavelength of 532 nm.

For WB analysis, EVs were lysed in RIPA buffer, and protein concentration was determined using a BCA Protein Assay Kit (Art. No. A53226; Thermo Fisher Scientific, USA). EV marker proteins, including CD63 (ab216130, 1:1,000) and HSP70 (ab2787, 1:1,000), as well as the endoplasmic reticulum marker calnexin (ab22595, 1:1,000), were detected by WB. All antibodies were purchased from Abcam (UK). Each experiment was performed in triplicate.

### Immunofluorescence

Cells were seeded onto sterile 22 × 22 mm glass coverslips and cultured for 24 h to allow attachment. The cells were then stimulated with 100 ng/mL EGF for the indicated time periods. After treatment, the cells were washed twice with PBS, fixed with 4% paraformaldehyde for 20 min at RT, and permeabilized with 0.25% Triton X-100 for 10 min. After two additional washes with PBS, the cells were blocked with 3% bovine serum albumin (BSA) for 1 h at RT to reduce nonspecific binding.

The cells were then incubated with the indicated primary antibodies overnight at 4 °C. On the following day, the cells were washed three times with PBS and incubated with fluorophore-conjugated secondary antibodies for 1 h at RT in the dark. After three additional washes with PBS, nuclei were counterstained with DAPI for 15 min. Coverslips were mounted, and fluorescence images were acquired using a confocal laser scanning microscope (FV3000; Olympus, Japan). Images were exported and analyzed using Fiji ImageJ software [46].

### Uptake of PKH26-labeled GC9811-P-derived EVs by GC cells

Isolated GC9811-P-EVs were incubated with the red fluorescent dye PKH26 (Art. No: PKH26GL-1KT, Sigma-Aldrich, USA) at RT for 5 min. After labeling, the EVs were washed with PBS and ultracentrifuged at 100,000 × g for 90 min. The labeled EV pellet was then resuspended in basal medium and co-cultured with MKN74 or AGS cells at a final concentration of 10 µg/mL for 24 h at 37 °C. After incubation, the cells were fixed with 4% paraformaldehyde, and nuclei were stained with Hoechst 33,342 (C1025; Beyotime, Nantong, China) at 10 µg/mL for 10 min. EV uptake by MKN74 and AGS cells was observed under a fluorescence microscope (ECLIPSE E800; Nikon, Japan).

### Cell infection and grouping

Gene overexpression and knockdown were achieved by lentiviral transduction. Lentiviral packaging was performed by ShengGong Bioengineering (Shanghai, China). Briefly, pHAGE-puro series plasmids together with the packaging plasmids pSPAX2 and pMD2.G, or pSuper-retro-puro series plasmids together with gag/pol and VSVG helper plasmids, were co-transfected into 293T cells (CRL-3216; ATCC, USA). After 48 h of culture, the supernatant containing lentiviral particles was collected, passed through a 0.45-µm filter, and concentrated by centrifugation. Viral particles were harvested at 72 h, and viral titers were determined before use.

Cells in the logarithmic growth phase were digested with trypsin and seeded into 6-well plates at 1 × 10^5^ cells per well. After 24 h, cells were infected with lentivirus at a multiplicity of infection (MOI) of 10 (working titer, approximately 5 × 10^6^ TU/mL) in the presence of 5 µg/mL polybrene (TR-1003; Merck, USA). After 4 h, the medium was replaced with fresh complete medium. For stable cell line generation, puromycin (10 µg/mL; A100339; ShengGong Bioengineering, Shanghai, China) was used for selection. The sequences of the knockdown lentiviral constructs are listed in Table S2.

The grouping of MKN74 and GC9811-P cells is as follows: (1) mock group (normal culture); (2) sh-NC group (infected with silencing lentiviral vector); (3) sh-GALNT1-1 group (infected with GALNT1 silencing lentivirus-1); (4) sh-GALNT1-2 group (infected with GALNT1 silencing lentivirus-2); (5) sh-GALNT1 + oe-RPRD1A group (infected with GALNT1 silencing lentivirus and overexpressed RPRD1A lentivirus).

The grouping of AGS cells is as follows: (1) mock group (normal culture); (2) oe-NC group (infected with overexpressed lentiviral vector); (3) oe-GALNT1 group (infected with overexpressed GALNT1 lentivirus); (4) sh-NC group (infected with silencing lentiviral vector); (5) sh-RPRD1A-1 group (infected with RPRD1A-silencing lentivirus-1); (6) sh-RPRD1A-2 group (infected with RPRD1A-silencing lentivirus-2); (7) sh-GALNT1 + oe-RPRD1A group (infected with GALNT1 silencing lentivirus and overexpressed RPRD1A lentivirus).

For GC9811-P-EV treatment experiments, the groups were defined as follows: (1) PBS, cells treated with PBS; (2) GC9811-P-EVs, cells treated with EVs derived from GC9811-P cells; (3) GES-1-EVs, cells treated with EVs derived from the normal gastric epithelial cell line GES-1 as a source-specific control; (4) sh-NC-EVs, cells treated with EVs derived from GC9811-P cells transduced with control shRNA lentivirus; (5) sh-GALNT1-EVs, cells treated with EVs derived from GC9811-P cells transduced with GALNT1-silencing lentivirus; and (6) sh-GALNT1-EVs + oe-RPRD1A, RPRD1A-overexpressing GC cells co-cultured with EVs derived from GC9811-P cells transduced with GALNT1-silencing lentivirus.

To evaluate the effect of O-GalNAc glycosylation inhibition on the molecular weight of RPRD1A, cells were treated with 2 mM benzyl-α-GalNAc (Benzyl-α-GalNAc; HY-129389; MCE, Shanghai, China) for 24 h.

### RT-qPCR detection of relative expression levels of GALNT1 and RPRD1A

Total RNA was extracted from tissues and cells using Trizol (Art.No: 16096020, purchased from USA Thermo Fisher Scientific). The RNA concentration and purity were determined using the NanoDrop One/OneC microvolume nucleic acid protein analyzer from Thermo Scientific (A260/A280 = 2.0, concentration > 5 µg/µL). Complementary DNA (cDNA) was synthesized from total RNA using a first-strand cDNA synthesis kit (Art. No. D7168L; Beyotime, Shanghai, China).

RT–qPCR was performed using a commercial qPCR kit (Art. No. Q511-02; Vazyme Biotech, Nanjing, China). Each 20-µL reaction contained 2 µL of cDNA template, 0.2 µL each of forward and reverse primers, 10 µL of qPCR mix, and RNase-free water. Amplification was carried out on a CFX96 real-time PCR system (Bio-Rad, USA) under the following conditions: 95 °C for 30 s, followed by 40 cycles of 95 °C for 10 s, 60 °C for 30 s, and 72 °C for 30 s, with a final melting curve analysis from 65 °C to 95 °C. Primer sequences were designed and synthesized by Shanghai Sengen Biotech (Shanghai, China) and are listed in Table S3. Relative gene expression was calculated using the 2^−ΔΔCt^ method with β-actin as the internal reference. Each sample was analyzed in triplicate.

### Western blot

Total protein was extracted from tissues and cultured cells using RIPA lysis buffer supplemented with 1% PMSF (P0013B; Beyotime, Shanghai, China). Membrane proteins were isolated using a ProteoPrep^®^ Membrane Extraction Kit (ProTEM-1KT; Merck, Germany). Protein concentrations were determined using a BCA Protein Assay Kit (P0011; Beyotime, Shanghai, China). According to the molecular weight of the target proteins, 8%–12% SDS–PAGE gels were prepared, and equal amounts of protein were loaded into each lane for electrophoretic separation. Proteins were then transferred onto PVDF membranes (1620177; Bio-Rad, USA), which were blocked with 5% skim milk for 1 h at RT. Primary antibody (Table S4) was added and incubated at 4 ℃ overnight. After incubation, membranes were washed three times with 1× TBST for 5 min each and then incubated with HRP-conjugated goat anti-rabbit IgG (ab6721, 1:2,000) or goat anti-mouse IgG (ab6728, 1:2,000) secondary antibodies at RT. After three additional washes with 1× TBST, protein bands were visualized using ECL reagent (1705062; Bio-Rad, USA) and imaged with an ImageQuant LAS 4000 C imaging system (GE Healthcare, USA). β-actin was used as the internal control for total and cytoplasmic proteins. Relative protein expression was quantified by calculating the gray value ratio of the target band to the corresponding internal control band. All assays were performed in triplicate.

### Lectin pull-down assay

To assess changes in O-glycosylation, 300 µg of total cell lysate was incubated overnight at 4 °C with Vicia villosa agglutinin (VVA)-agarose beads (L-1230-5; Vector Laboratories, Burlingame, CA, USA). After two washes with PBS, the precipitated RPRD1A protein was analyzed by WB [47].

### Wound healing assay

For evaluation of cell migratory capacity, parallel reference lines were drawn on the underside of 6-well plates at intervals of 0.5–1 cm, with at least five lines crossing each well. Cells were seeded at 5 × 10^5^ cells per well and cultured overnight in complete medium. On the following day, a linear wound was created perpendicular to the reference lines using a 200-µL pipette tip. The medium was then replaced with serum-free medium. Wound widths were recorded at 0 h and 48 h under a light microscope (DM500; Leica, Germany), and images were acquired using an inverted microscope. Cell migration was evaluated by comparing wound closure at the indicated time points.

### Colony formation assay

For the colony formation assay, cells were dissociated into single-cell suspensions, and 1,000 cells were seeded into each 6-cm culture dish. Complete medium was added and replaced every 3 days. After 8–10 days of culture, the cells were washed with PBS, fixed with 4% paraformaldehyde, and stained with 0.4% crystal violet for 15 min. Colonies containing more than 10 cells were counted manually, and the average number of colonies was calculated from replicate dishes.

### Transwell invasion assay

Cell invasion was evaluated using Transwell chambers with 8-µm pore size inserts (Corning, USA) in 24-well plates. Matrigel-coated inserts were pre-equilibrated with DMEM containing 20% FBS in the lower chamber at 37 °C for 1 h. At 48 h after transfection, GC cells were resuspended in serum-free DMEM at a density of 1 × 10^6^ cells/mL, and the cell suspension was added to the upper chamber. After incubation at 37 °C in 5% CO_2_ for 24 h, the inserts were removed, washed twice with PBS, fixed with 5% glutaraldehyde, and stained with 0.1% crystal violet for 5 min. Non-invading cells on the upper surface were gently removed with a cotton swab. Invaded cells on the lower surface were observed under a microscope (TE2000; Nikon, Japan). Five random fields were photographed for each insert, and the average number of invaded cells was calculated. Each experiment was repeated three times.

### CCK-8 assay

Cell proliferation after co-culture with CD8^+^ T cells was assessed using a CCK-8 assay kit (40203ES60; Yeasen, Shanghai, China). Cells in the logarithmic growth phase were adjusted to 5 × 10^4^ cells/mL in complete medium, and 100 µL of cell suspension was seeded into each well of a 96-well plate. After 24 h of incubation, the medium was replaced with fresh complete medium containing 10 µL of CCK-8 reagent per well. The plate was further incubated at 37 °C for 2 h, and absorbance at 450 nm was measured using a Multiskan FC microplate reader (51119080; Thermo Fisher Scientific, USA). Each group contained three parallel wells, and all experiments were repeated three times [48, 49].

### Immunofluorescence staining

Cells were cultured on coverslips (SPL Life Sciences, Gyeonggi-do, Korea), fixed with 4% PFA, and permeabilized with 0.25% Triton X-100. After blocking in PBS containing 2% BSA (Bio-Rad), the cells were incubated with primary antibodies against RPRD1A (Table S4). Isotype-matched antibodies were used as controls. A Goat Anti-Rabbit IgG H&L (Alexa Fluor^®^ 488) secondary antibody (ab150077, Abcam, UK) was used. Cell nuclei were visualized with DAPI (SD3571, Thermo Fisher, USA). Images were captured using a fluorescence microscope (ECLIPSE E800, Nikon, Japan) and analyzed using Photoshop 5.0 software [50, 51].

### Flow cytometry analysis

Cells were washed with PBS and resuspended at 1 × 10^7^ cells/mL in 100 µL PBS per sample. For intracellular staining, cells were permeabilized with 0.5% Tween 20 (P2287; Merck KGaA, Germany) for 5 min and then incubated with the indicated antibodies at 4 °C. Flow cytometry was performed using a BD LSRFortessa instrument (BD Biosciences, USA). FITC-conjugated anti-CD45 (103108; BioLegend, Canada) and PerCP-Cyanine5.5-conjugated anti-CD8 (45-0081-82; Invitrogen, Thermo Fisher Scientific, Shanghai, China) were used. Data were analyzed with BD FACSDiva software [52].

### Preparation of LNPs@GALNT1 siRNA

To achieve stable delivery of GALNT1-targeted siRNA and enhance its uptake efficiency in GC cells, LNPs@GALNT1 siRNA were constructed. The main materials included DSPE-PEG2000 and DSPE-PEG2000-Mal (Avanti Polar Lipids, Alabaster, AL, USA), stearic acid (SA) and polyethyleneimine (PEI1800) (Aladdin, Shanghai, China), EDC and NHS (J&K Scientific, Beijing, China), GALNT1 siRNA (GenePharma, Shanghai, China), and EpCAM monoclonal antibody (ab71916, Abcam, UK).

First, 142.4 mg of SA was dissolved in 10 mL of DMSO, followed by the addition of 115 mg EDC and 69 mg NHS to activate the carboxyl groups. After magnetic stirring at RT for 2 h, 1504.2 mg PEI1800 was added, and the reaction was allowed to proceed for 24 h. The reaction mixture was extracted with ethyl acetate to remove unreacted SA, dialyzed against distilled water for 48 h, and lyophilized to obtain the PSA polymer.

For antibody conjugation, the EpCAM antibody and DSPE-PEG2000-Mal were mixed at a molar ratio of 1:1.5 in chloroform/methanol (2:1, v/v) and reacted at RT for 48 h in the presence of triethylamine as a catalyst. The reaction endpoint was monitored by thin-layer chromatography, and the product was purified by dialysis and collected after lyophilization.

For nanoparticle assembly, DSPE-PEG2000, DSPE-PEG2000-EpCAM, and PSA were dissolved in methanol at a weight ratio of 8:2:1. The solvent was removed by rotary evaporation (RE-2000 A; Yarong, Shanghai, China) under reduced pressure to form a lipid film. The film was hydrated with PBS (pH 7.4) for 20 min and sonicated using a probe sonicator (SCI-ENTZ18-A; Ningbo Xinzhi, China) at 80 W for 2 min to generate micelles.

GALNT1 siRNA was then mixed with the micelles at a weight ratio of 1:5, vortexed for 15 s, and incubated at RT for 15 min to form LNPs@GALNT1 siRNA nanoparticles [53].

### Characterization of LNPs@GALNT1 siRNA

The physicochemical properties and stability of LNPs@GALNT1 siRNA were characterized by DLS and TEM. Mean particle size and zeta potential were measured using a Zetasizer Nano ZS90 instrument (Malvern Instruments, UK). LNPs were diluted to 1 mg/mL, transferred into plastic cuvettes, and measured in triplicate at 25 °C. Particle morphology was examined by TEM (Tecnai G2 Spirit BioTWIN; FEI, USA). Samples were diluted 10-fold with deionized water, placed onto carbon-coated copper grids (Electron Microscopy Sciences, USA), air-dried for 10 min, negatively stained with 2% phosphotungstic acid (Sigma-Aldrich, USA) for 30 s, and then imaged.

For serum stability analysis, LNPs@GALNT1 siRNA were incubated in PBS containing 10% FBS at 37 °C in a shaking water bath for 72 h. Particle size was measured by DLS at 0, 6, 12, 24, 48, and 72 h. For storage stability analysis, LNP suspensions were stored at 4 °C, and particle size was monitored for 7 days. Zeta potential stability was further assessed after incubation at 37 °C for 72 h. The siRNA protection capability was evaluated by incubating LNPs@GALNT1 siRNA or an equivalent amount of free GALNT1 siRNA in PBS containing 10% FBS at 37 °C for 24 h. Samples were collected at 0, 1, 2, 4, 8, 12, 16, 20, and 24 h and treated with 1% SDS (Sigma-Aldrich, USA) to disrupt the nanoparticles. siRNA integrity was then analyzed by 1% agarose gel electrophoresis (Bio-Rad, USA).

### Biosafety assessment of LNPs@GALNT1 siRNA

To systematically evaluate the in vivo biosafety of LNPs@GALNT1 siRNA, male C57BL/6 mice aged 6–8 weeks (strain code: 219; Beijing Vital River Laboratory Animal Technology Co., Ltd., China) were used. The mice received intravenous injections of LNPs@GALNT1 siRNA via the tail vein at a dose of 100 nmol/kg body weight. After 7 days of treatment, the animals were sacrificed, and major organs (heart, liver, spleen, lungs, and kidneys) as well as blood samples were collected. All animal experiments were approved by the Animal Ethics Committee of Beijing Vital River Laboratory Animal Technology Co., Ltd.

Collected tissues were fixed in 4% neutral formalin (Sigma-Aldrich, USA) for 48 h, embedded in paraffin, sectioned, and subjected to hematoxylin and eosin (H&E) staining (Solarbio, China) for histopathological evaluation. Tissue morphology was examined under a light microscope (BX53; Olympus, Japan). Hematological parameters, including red blood cell count (RBC), white blood cell count (WBC), hemoglobin concentration (HGB), and platelet count (PLT), were measured using an automated hematology analyzer (Sysmex XN-1000, Sysmex, Japan). Serum was separated by centrifugation (Eppendorf 5810R, Germany), and the alanine aminotransferase (ALT), aspartate aminotransferase (AST), blood urea nitrogen (BUN), and creatinine (CRE) levels were analyzed using an automated biochemical analyzer (Cobas c701, Roche Diagnostics, Switzerland).

These analyses were used to comprehensively evaluate the hematological, biochemical, and histopathological safety of the LNP formulation.

### In vivo distribution analysis of LNPs@GALNT1 siRNA

To evaluate the biodistribution and tumor accumulation profile of LNPs@GALNT1 siRNA, a subcutaneous GC xenograft mouse model was established. Cy5.5-labeled free GALNT1 siRNA or Cy5.5-labeled LNPs@GALNT1 siRNA was administered via tail vein injection. In vivo fluorescence imaging was performed at 6, 12, and 24 h after injection to monitor systemic distribution and tumor-associated fluorescence signals. Free Cy5.5-labeled GALNT1 siRNA showed rapid clearance and limited tumor accumulation, whereas Cy5.5-labeled LNPs@GALNT1 siRNA exhibited sustained fluorescence enrichment in the tumor region, suggesting improved in vivo stability and enhanced tumor accumulation. Tumor tissues were further collected for ex vivo fluorescence imaging and quantitative analysis to assess intratumoral siRNA accumulation.

Each mouse was injected subcutaneously in the right hind limb with 5 × 10⁶ MKN74 cells. When the tumor diameter reached 5 mm, Cy5.5-labeled GALNT1 siRNA or LNPs@GALNT1 siRNA (100 nmol/kg; Cy5.5-NHS Ester from Lumiprobe, USA) was administered via tail vein injection. Fluorescence images were acquired at 6, 12, and 24 h post-injection using the IVIS Spectrum 2 in vivo imaging system (PerkinElmer, USA), and analyzed with Living Image software to assess fluorescence distribution and signal intensity within the tumor region.

At 12 h after injection, tumor tissues were harvested, embedded in OCT compound (Sakura, USA), and sectioned into 10-µm frozen slices. Nuclei were counterstained with DAPI, and Cy5.5 fluorescence in tumor sections was examined by confocal microscopy. At 6, 12, and 24 h after injection, the heart, liver, spleen, lungs, kidneys, and tumors were collected for ex vivo fluorescence imaging using the IVIS system. Fluorescence intensity in each organ was quantified to evaluate the in vivo distribution and tumor-targeting efficiency of the LNP formulation [54].

### Construction of HIS mice

HIS mice were generated using 4-week-old female NSG (NOD-Prkdc^scid^Il2rg^tm1/Bcgen^) mice (Art. No. NM-NSG-001; Shanghai Southern Model Biological Technology Co., Ltd., Shanghai, China). Mice were conditioned with 2.4 Gy γ-irradiation for bone marrow ablation, followed by transplantation of human CD34^+^ hematopoietic stem cells (hCD34^+^ HSCs). Briefly, mononuclear cells were isolated from human umbilical cord blood (hUCB) by Ficoll-Paque Plus density-gradient centrifugation, and hCD34^+^ cells were enriched using a human CD34 MicroBeads kit and magnetic-activated cell sorting (Miltenyi Biotec, Spain). The purified hCD34^+^ cells were intravenously injected into irradiated mice within 24 h after myeloablation. Cell purity was assessed using a CytoFLEX flow cytometer. Successful human immune reconstitution was defined as human CD45^+^ cells accounting for at least 25% of peripheral blood mononuclear cells (Figure S3A-B).

### Establishment of GC in vivo mouse models

The previously established HIS mice were subcutaneously injected with 1 × 10^7^ cells/0.2 ml of GC9811-P or MKN74 cells, divided into the following groups based on the injected material: sh-NC group, sh-GALNT1 group, sh-GALNT1 + oe-RPRD1A group. For peritoneal metastasis experiments, the above cells (5 × 10^6^ cells) were injected into the mouse peritoneal cavity to observe peritoneal metastasis of MKN74 and GC9811-P. Each group consisted of 6 mice.

To assess the effects of GC9811-P-derived EVs in vivo, 1 × 10^7^ transfected or non-transfected MKN74 cells in 0.2 mL were subcutaneously injected into HIS mice. One week later, mice received tail-vein injections of EVs at 5 µg/day according to the experimental design. The EV treatment groups were defined as PBS, GC9811-P-EVs, sh-NC-EVs, and sh-GALNT1-EVs + oe-RPRD1A. For peritoneal metastasis experiments, 5 × 10^6^ MKN74 cells were injected intraperitoneally, followed 1 week later by tail-vein administration of the indicated EVs at 5 µg/day. Each group contained six mice.

To evaluate the antitumor efficacy and immunomodulatory effects of LNPs@GALNT1 siRNA, subcutaneous and peritoneal metastasis models were also established in HIS mice. Animals were randomly assigned to the PBS, GALNT1 siRNA, and LNPs@GALNT1 siRNA groups, with six mice per group. Mice in the treatment groups received tail-vein injections of free GALNT1 siRNA or LNPs@GALNT1 siRNA at 100 nmol/kg every 2 days for a total of five injections.

Tumor growth was monitored weekly using calipers. The short diameter (a) and long diameter (b) were recorded, and tumor volume was calculated as π*(a^2^b)/6. Tumor weight was measured at the study endpoint. After 6 weeks, mice were euthanized, and tumors and ascites were collected. Half of each tumor sample was snap-frozen in liquid nitrogen, and the remaining half was processed for histological analysis.

### CD8⁺ T-cell proliferation assay

CD8^+^ T-cell proliferation was assessed using a CFSE Cell Proliferation Kit (BB-4701, BestBio, China). Briefly, isolated CD8^+^ T cells were labeled with CFSE at a final concentration of 4 µmol/L, with 1 × 10^7^ cells stained per reaction as recommended by the kit protocol. After labeling, the cells were cultured under the indicated conditions. The percentage of CFSE-positive cells and the changes in fluorescence intensity were then analyzed by flow cytometry (BD Biosciences, San Jose, CA, USA). CD8⁺ T-cell proliferation was evaluated based on the progressive dilution of CFSE fluorescence during successive cell divisions [55].

### Immunohistochemical staining

Tissue samples were fixed in formalin, embedded in paraffin, and sectioned at 4 μm thickness. After deparaffinization and rehydration, immunohistochemical staining was performed according to standard procedures. The primary antibodies used were rabbit anti-GALNT1 (ab253025, 1:200; Abcam, UK), rabbit anti-RPRD1A (NBP1-87917, 1:500; Bio-Techne, USA), rabbit anti-CD8 (ab209775, 1:200; Abcam, UK), and rabbit anti-CD4 (ab213215, 1:200; Abcam, UK). Sections were incubated with primary antibodies overnight at 4 °C, followed by incubation with goat anti-rabbit IgG secondary antibody (ab6721, 1:1,000; Abcam, UK) for 30 min. The streptavidin-biotin complex (SABC; Vector Laboratories, USA) was then applied at 37 °C for 30 min. Immunoreactivity was visualized using a DAB kit (P0203; Beyotime Biotechnology, Shanghai, China), and nuclei were counterstained with hematoxylin for 30 s. Sections were subsequently dehydrated through graded ethanol, cleared in xylene, and mounted with neutral resin. Images were acquired using an upright microscope (BX63; Olympus, Japan). The proportion of positive cells was quantified by grayscale analysis using ImageJ software.

### ELISA detection of immune factor expression

Cell culture supernatants or mouse serum samples were centrifuged at 1,509.3 × g for 15 min before analysis. IFN-γ, TNF-α, and granzyme B (Gzmb) levels were measured using commercial ELISA kits according to the manufacturers’ instructions: IFN-γ (MIF00; R&D Systems, USA), TNF-α (ab208348; Abcam, UK), and Gzmb (BMS2027-2TEN; Invitrogen, USA). Absorbance was recorded at 450 nm using an Epoch microplate spectrophotometer (BioTek, Winooski, VT, USA) [56]. Each sample was assayed in triplicate [57].

### Statistical analysis

Data were obtained from three independent experiments and presented as mean ± standard deviation (Mean ± SD). For comparisons between the two groups, we used a two-sample independent t-test. A one-way analysis of variance (ANOVA) was employed for comparisons among three or more groups. Repeated measures analysis of variance was used to compare differences between data at different time points. If the ANOVA results showed significant differences, Tukey’s Honestly Significant Difference (HSD) post hoc test was conducted to compare differences between groups. For non-normally distributed or heteroscedastic data, the Mann–Whitney U test or Kruskal–Wallis test was applied. All statistical analyses were performed using Origin 9.0 and R software. *p <* 0.05 indicated statistical significance.

## Results

### EVs derived from high metastatic, peritoneal GC cells, promote immune escape and peritoneal metastasis

To further characterize the TME associated with peritoneal metastasis in GC, we first examined a panel of GC cell lines, including AGS, SNU-520, FU97, SNU-719, MKN74, and GC9811-P cells. To identify an appropriate highly metastatic model for subsequent mechanistic studies, the migratory and invasive capacities of these cell lines were assessed by wound-healing and Transwell assays. AGS cells exhibited the weakest metastatic potential, whereas MKN74 and GC9811-P cells showed markedly stronger migratory and invasive abilities, with GC9811-P cells displaying the highest metastatic capacity (Figure S1A-B). TEM, DLS, and WB analyses further confirmed that EVs isolated from GC9811-P cells displayed the characteristic cup-shaped morphology and bilayer membrane structure of EVs, with particle diameters ranging from 30 to 100 nm. These EVs were positive for the EV markers CD63 and HSP70, but negative for the endoplasmic reticulum marker calnexin (Figure S1C-E).

**Fig. 1:**
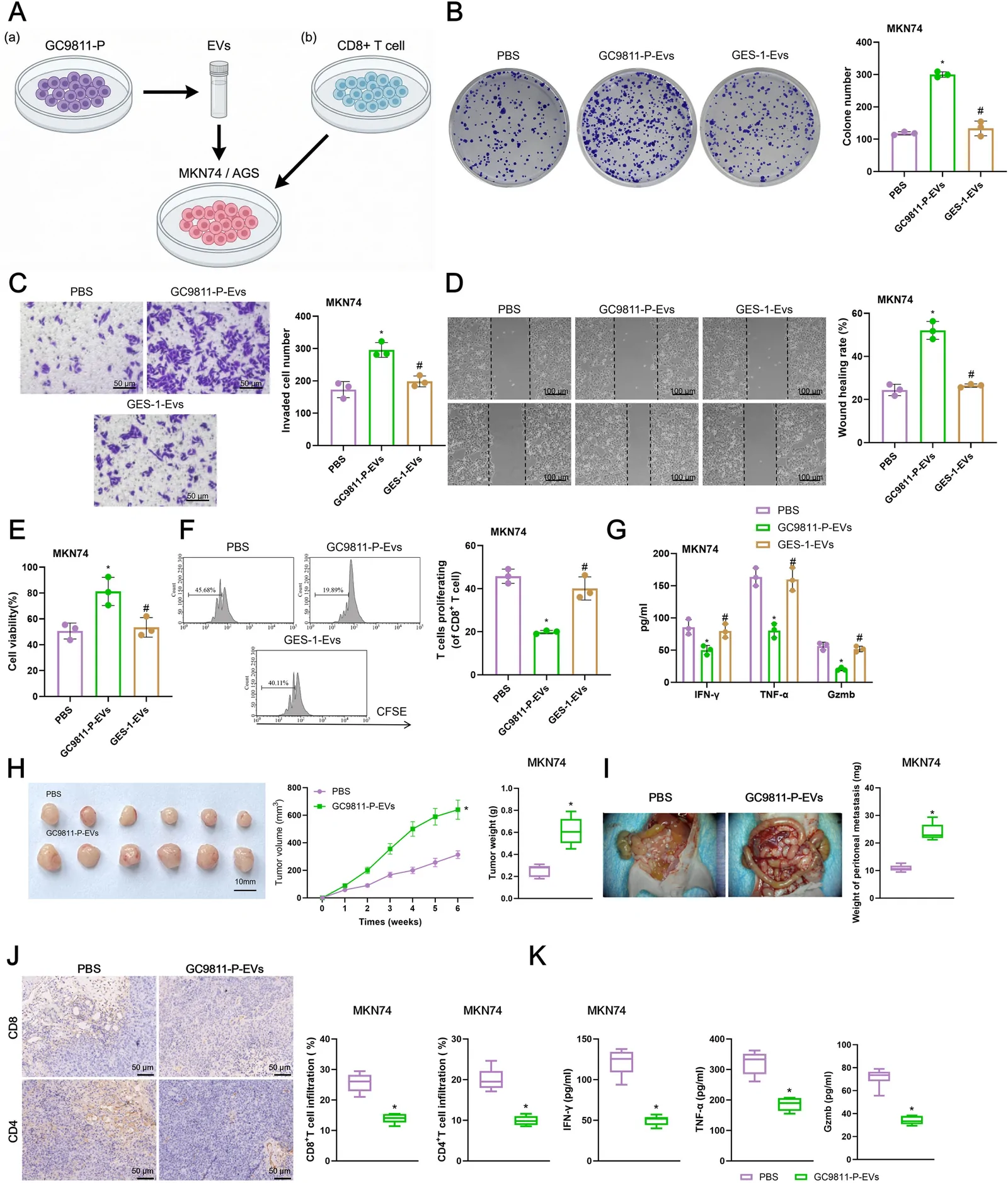
Effect of GC9811-P-EVs on immune escape and peritoneal metastasis of GC cells. Note: **A** Flow chart of in vitro co-culture experiment; **B** Clonal formation assay was used to detect cell proliferation ability in each group. **C** The invasion ability of cells in each group was detected by Transwell assay (scale bar = 50 μm). **D** The metastasis ability of cells in each group was detected by scratch test. **E** Proliferation capacity of tumor cells after co-culture with CD8^+^T cells; **F** Proliferation of CD8⁺ T cells after coculture, as determined by the CFSE Cell Proliferation Kit; **G **The levels of immune-related factors IFN-γ, TNF-α, and Gzmb in the co-culture supernatant were detected by ELISA. **H** Tumor growth curve, representative pictures of tumor bodies and tumor weight statistics of mice in each group; **I** The mesenteric tumors were observed and weighed in each group. **J** The infiltration of CD8^+^ and CD4^+^T cells in mesenteric tumors in each group was detected by immunohistochemical staining (scale bar = 50 μm). **K** ELISA was used to detect the expression of immune-related factors IFN-γ, TNF-α and Gzmb in ascites. *p < 0.05 versus the PBS group; #*p <* 0.05 versus the GC9811-P-EVs group. Cell experiments were repeated three times. Six mice were included in each group

Because immunosuppressive cells and cytokines cooperatively shape an immunosuppressive TME and contribute to CD8^+^ T-cell dysfunction [58], we next investigated whether GC9811-P-derived EVs modulate the immune microenvironment. GC9811-P-EVs were co-cultured with low-metastatic AGS cells and highly metastatic MKN74 cells (Fig. 1A). Fluorescence imaging showed that both AGS and MKN74 cells efficiently internalized GC9811-P-derived EVs (Figure S1F). Compared with PBS, GC9811-P-EVs significantly enhanced the proliferation, migration, and invasion of both AGS and MKN74 cells (Fig. 1B-D, Figure S2A-C). In contrast, the protumorigenic effects of GES-1-derived EVs were substantially weaker than those of GC9811-P-EVs. We then evaluated whether GC9811-P-EVs affected antitumor T-cell activity. CD8^+^ T cells were co-cultured with AGS and MKN74 cells pretreated with GC9811-P-EVs (Fig. 1A). GC9811-P-EVs significantly attenuated T-cell-mediated tumor cell killing compared with PBS treatment (Fig. 1E, Figure S2D). Consistently, CFSE-based proliferation assays showed that CD8^+^ T-cell proliferation was markedly reduced after exposure to GC9811-P-EVs. ELISA further demonstrated that the levels of IFN-γ, TNF-α, and Gzmb in the co-culture supernatants were significantly decreased. By comparison, GES-1-EVs exerted much weaker inhibitory effects on T-cell function (Fig. 1F-G, Figure S2E-F).

To further validate these findings in vivo, we established HIS mice by transplanting hCD34^+^ hematopoietic stem cells into NSG mice. At 13 weeks after transplantation, flow cytometric analysis of peripheral blood showed successful immune reconstitution, as indicated by the proportion of hCD45⁺ cells (Figure S3A-B).

MKN74 cells were then inoculated subcutaneously into HIS mice, followed by weekly tail-vein injection of GC9811-P-EVs. Compared with the PBS group, mice treated with GC9811-P-EVs exhibited significantly increased tumor growth and tumor weight (Fig. 1H).

To determine whether GC9811-P-EVs also promote peritoneal dissemination, we established a peritoneal metastasis model by intraperitoneal injection of MKN74 cells. Four weeks later, mesenteric metastatic tumors were collected and weighed. GC9811-P-EV treatment significantly increased both the metastatic burden and tumor weight compared with PBS treatment (Fig. 1I). Because reduced activation and infiltration of CD4^+^ and CD8^+^ T cells represent important mechanisms of tumor immune escape [58], we next assessed tumor-infiltrating lymphocytes by immunohistochemistry. GC9811-P-EV treatment markedly reduced intratumoral CD4^+^ and CD8^+^ T-cell infiltration relative to the PBS group (Fig. 1J). In addition, ELISA showed that the levels of IFN-γ, TNF-α, and Gzmb in ascites were significantly decreased in mice treated with GC9811-P-EVs (Fig. 1K).

In summary, we found that GC9811-P-derived EVs promote immune escape and peritoneal metastasis of GC cells.

### Integrated bioinformatic and clinical analyses identify the GALNT1/RPRD1A axis as a potential core pathway in GC immune escape

Aberrant glycosylation affects multiple cellular processes, including proliferation, apoptosis, differentiation, adhesion, migration, invasion, and immune responses [26]. Among these modifications, GalNAc-type O-glycosylation and its initiating enzymes, the GALNT family, play critical roles in regulating diverse cellular behaviors. In humans, this family comprises at least 20 members, designated GALNT1 to GALNT20 [59, 60]. To investigate the relevance of GALNT family genes to the immune microenvironment of GC, we first analyzed TCGA-STAD transcriptomic data and identified differential expression patterns among the 20 GALNT genes, including six upregulated and four downregulated members (Figure S4A-B). To further clarify the mechanism by which GC9811-P-derived EVs promote immune escape and peritoneal metastasis, we next examined the distribution of the six highly expressed GALNTs in GC-derived EVs using the exoRBase database. Among these candidates, GALNT1 showed the highest abundance in GC-derived EVs (Figure S4C). WB analysis further confirmed high GALNT1 expression in GC9811-P-EVs (Figure S4D). Previous studies have also shown that GALNT1 enhances the malignant phenotype of GC cells [29]. In addition, CIBERSORT and TIMER analyses revealed that GALNT1 expression was significantly negatively correlated with the infiltration of multiple T-cell subsets in GC (Figure S5A, B), suggesting a potential role for GALNT1 in tumor immune escape. We then explored the potential downstream regulatory network of GALNT1. A total of 30 candidate genes significantly associated with GALNT1 were identified, and a GALNT1-related protein interaction network was constructed (Figure S4E). Among these genes, RPRD1A showed not only a direct protein interaction with GALNT1, but also one of the strongest correlations with GALNT1 expression (Figure S4F). Moreover, RPRD1A expression was likewise negatively correlated with the infiltration of multiple T-cell subsets in GC (Figure S5C, D), indicating that it may participate in GALNT1-mediated immunosuppressive regulation.

**Fig. 2:**
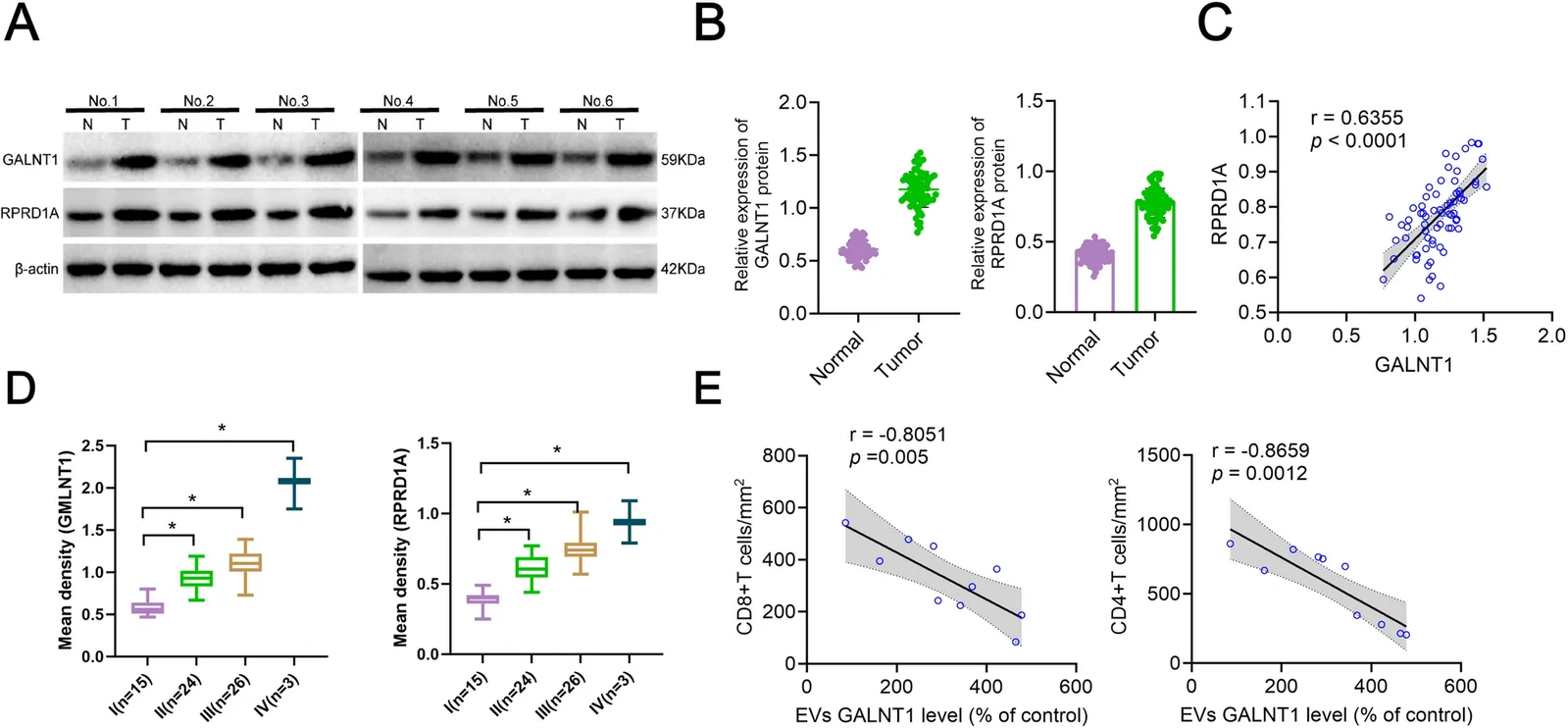
Expression levels of GALNT1 and RPRD1A in GC patients and cells. Note: **A**-**B** Western blot analysis of the expression levels of GALNT1 and RPRD1A in paired GC tissues and adjacent normal tissues of 68 cases. **C** Correlation between GALNT1 expression and RPRD1A expression in GC patients by Spearman analysis (*n* = 68); **D** The expression of GALNT1 and RPRD1A in cancer tissues of GC patients with different stages was detected by immunohistochemical staining (scale bar = 50 μm). **E** Correlation analysis between GALNT1 expression level of human GC-derived EVs and the number of infiltrated CD8^+^ T cells and CD4^+^ T cells per unit area (*n* = 10). (* indicates *p <* 0.05)

To validate these observations clinically, we collected 68 pairs of GC tissues and matched adjacent normal tissues and examined GALNT1 and RPRD1A expression. Compared with adjacent normal tissues, GC tissues showed significantly higher protein levels of both GALNT1 and RPRD1A (Fig. 2A, B), and their expression levels were positively correlated (Fig. 2C). Immunohistochemical staining further showed that GALNT1 and RPRD1A expression increased progressively with advancing clinical stage (Fig. 2D). When patients were stratified into high- and low-expression groups according to the median expression levels of GALNT1 and RPRD1A, elevated expression of either molecule was significantly associated with larger tumor size, more advanced tumor stage, and a higher frequency of lymph node metastasis (Table S5). Kaplan–Meier survival analysis further showed that high GALNT1 and RPRD1A expression was associated with poorer 5-year overall survival (Figure S4G). Consistent with the clinical findings, GALNT1 and RPRD1A expression was elevated in GC cell lines compared with GES-1 cells and was further increased in the highly metastatic MKN74 and GC9811-P cells (Figure S4H). To further examine the relationship between GC-derived EVs and immune infiltration, EVs were isolated from 10 patients with highly metastatic GC. Correlation analysis showed that GALNT1 expression in patient-derived EVs was negatively correlated with CD4^+^ and CD8^+^ T-cell infiltration in matched tumor tissues (Fig. 2E).

Collectively, these findings indicate that GALNT1 and RPRD1A are upregulated in GC, particularly in highly metastatic GC cells, and identify the GALNT1/RPRD1A axis as a potential core pathway associated with immune escape in GC.

### GALNT1 increases RPRD1A protein abundance to enhance the metastatic ability of GC cells

**Fig. 3:**
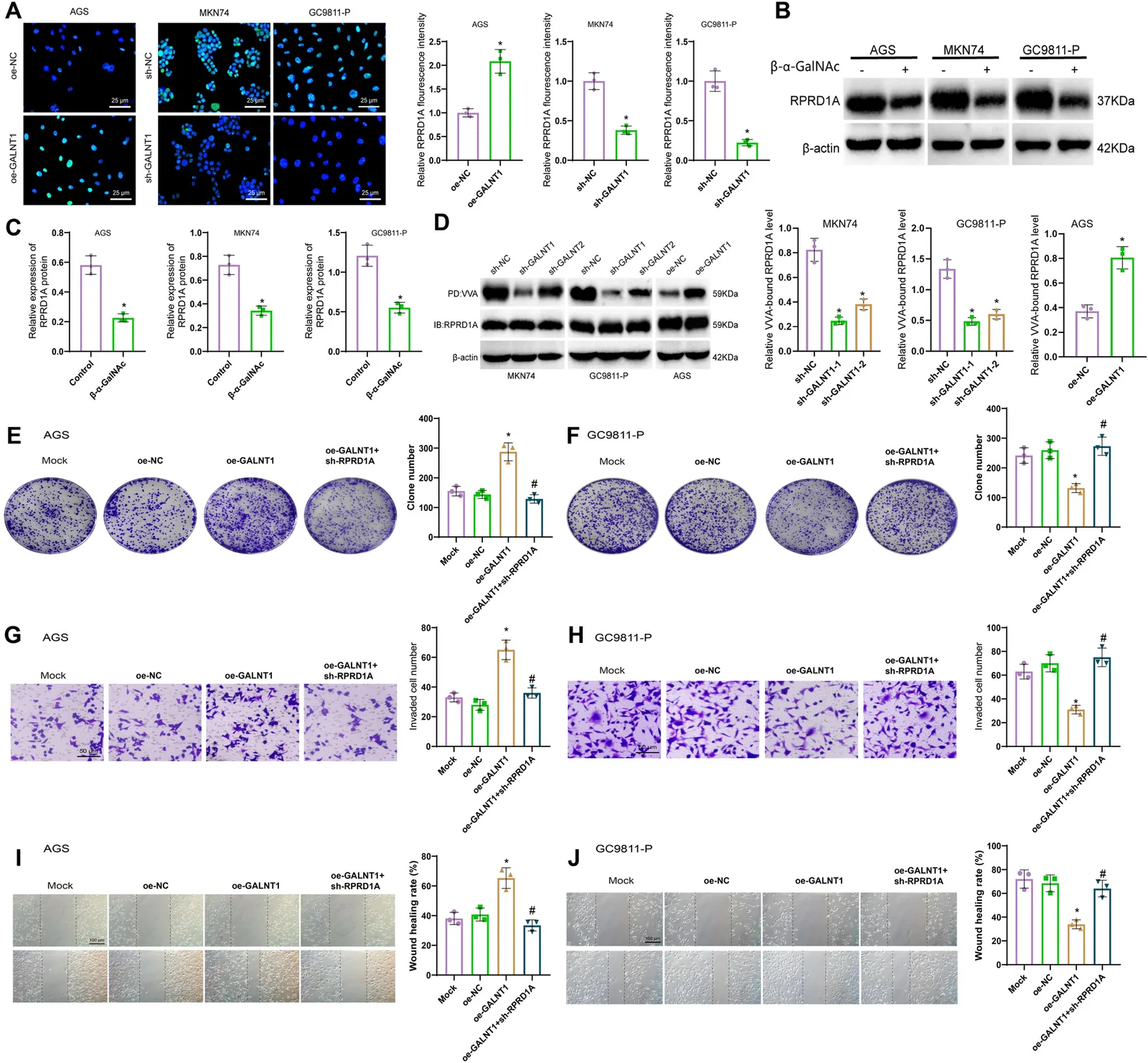
Effect of regulation of RPRD1A expression by GALNT1 on metastasis ability of highly metastatic GC cells. Note: **A** Confocal imaging of RPRD1A in GC cells. Cells were stained with anti-RPRD1A antibodies and FITC-conjugated secondary antibodies; nuclei were stained with DAPI. Scale bar = 25 μm. Relative RPRD1A fluorescence intensity was quantified from five fields per group; **B**-**C** Western blot analysis of RPRD1A molecular-weight changes after treatment with 2 mM benzyl-α-GalNAc (B-α-GalNAc) for 24 h; **D** VVA pull-down assay showing GalNAc-modified RPRD1A detected by Western blot; **E**-**F** Colony formation assays; **G**-**H** Transwell invasion assays (scale bar = 50 μm); **I**-**J** Wound-healing assays. *p < 0.05 compared with the sh-NC/oe-NC group; #*p <* 0.05 compared with the oe-GALNT1/sh-GALNT1 group. Cell experiments were repeated three independent times

As a glycosyltransferase, GALNT1 initiates mucin-type O-glycosylation and has been reported to promote protein glycosylation and activation [27, 61]. We further investigated whether GALNT1 regulates RPRD1A expression in GC cells. GALNT1 was silenced in MKN74 and GC9811-P cells and overexpressed in AGS cells, whereas RPRD1A was silenced in AGS cells and overexpressed in MKN74 and GC9811-P cells. Constructs with optimal knockdown efficiency were selected for subsequent experiments. RT-qPCR analysis showed that neither GALNT1 knockdown nor GALNT1 overexpression significantly altered RPRD1A mRNA levels (Figure S6B). In contrast, RPRD1A protein expression changed markedly at the protein level. GALNT1 overexpression increased RPRD1A protein expression in AGS cells, whereas GALNT1 silencing reduced RPRD1A protein levels in MKN74 and GC9811-P cells; this effect was reversed by RPRD1A overexpression (Figure S6D-E). Confocal imaging further confirmed that GALNT1 silencing markedly reduced RPRD1A expression in GC9811-P and MKN74 cells, whereas GALNT1 overexpression increased RPRD1A expression in AGS cells (Fig. 3A). These results suggest that GALNT1 regulates RPRD1A mainly at the protein level rather than the mRNA level.

To determine whether RPRD1A undergoes GalNAc-type O-glycosylation, GC cells were treated with benzyl-α-GalNAc (B-α-GalNAc), an inhibitor of O-glycan elongation. B-α-GalNAc treatment reduced the apparent molecular weight of RPRD1A in AGS, MKN74, and GC9811-P cells (Fig. 3B-C), indicating that RPRD1A carries O-glycans. To further examine whether GALNT1 was associated with this modification, we performed a Vicia villosa agglutinin (VVA) pull-down assay, as VVA specifically recognizes GalNAc residues on serine or threonine residues. Silencing GALNT1 in GC9811-P and MKN74 cells or reducing GALNT1 expression in AGS cells significantly decreased the GalNAc signal on RPRD1A (Fig. 3D). These results support GALNT1-dependent O-GalNAc modification of RPRD1A in GC cells.

We next assessed the biological consequences of this regulation. In AGS cells, GALNT1 overexpression significantly enhanced cell proliferation, migration, and invasion compared with the oe-NC group, whereas these effects were attenuated by RPRD1A knockdown (Fig. 3E, G, I). In MKN74 and GC9811-P cells, GALNT1 silencing significantly suppressed proliferation, migration, and invasion relative to the sh-NC group, while RPRD1A overexpression partially restored these malignant phenotypes (Fig. 3F, H, J, Figure S7).

Collectively, these results suggest that GALNT1 promotes the metastatic potential of GC cells by enhancing O-GalNAc modification and protein abundance of RPRD1A.

### GALNT1/RPRD1A axis promotes immune evasion of GC cells and enhances their metastatic abilities

**Fig. 4:**
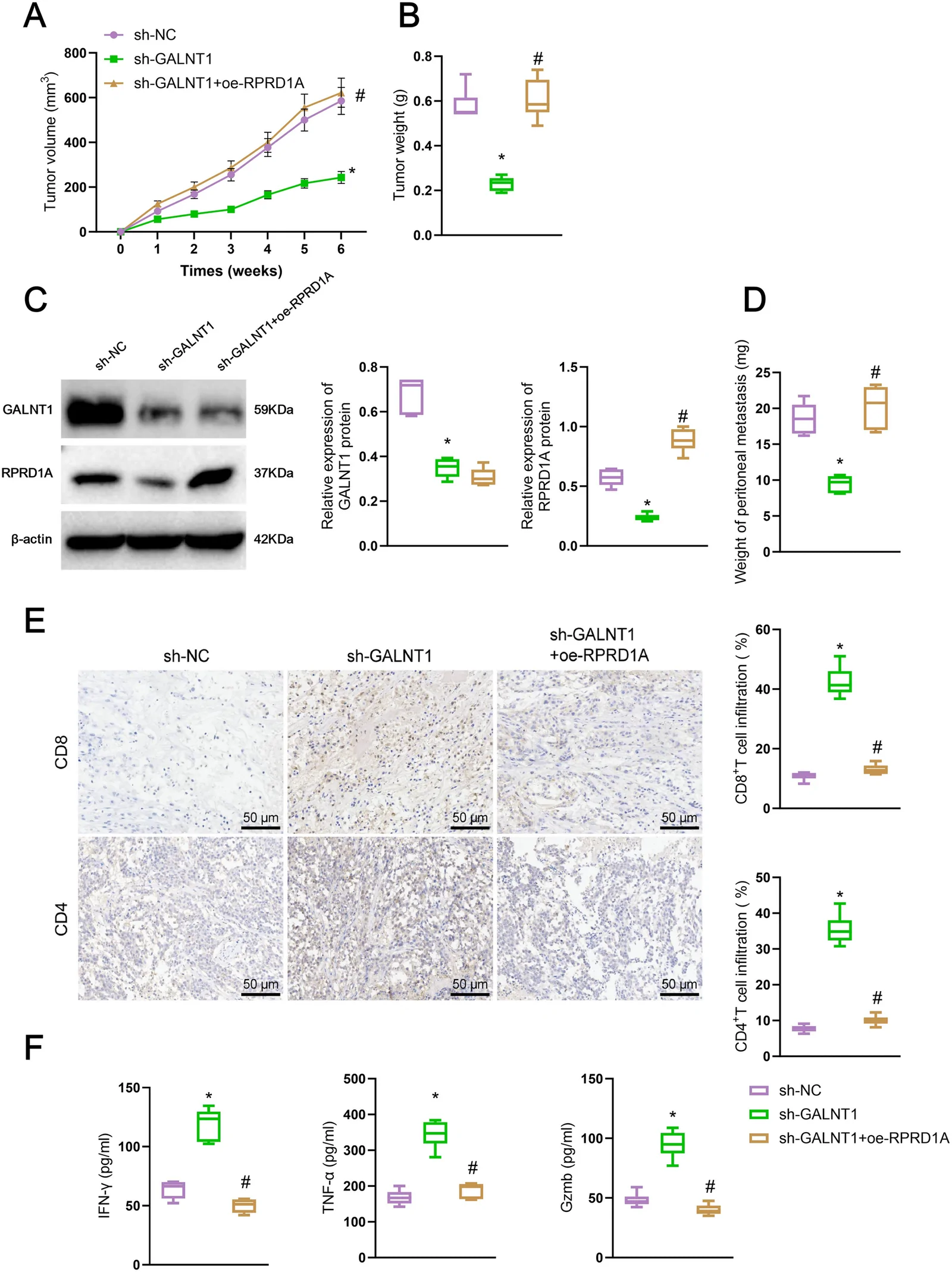
Effects of regulation of RPRD1A expression by GALNT1 on immune escape and metastasis of GC cells. Note: **A** Tumor growth curve of mice in each group; **B** Representative pictures of tumor bodies and tumor weight statistics in each group; **C** The expression of GALNT1 and RPRD1A in the tumor of mice in each group was detected by Western blot. **D** The mesenteric tumors were observed and weighed in each group. **E** The infiltration of CD8^+^ and CD4^+^T cells in mesenteric tumors in each group was detected by immunohistochemical staining (scale bar = 50 μm). **F** The levels of immune-related factors IFN-γ, TNF-α, and Gzmb in ascites were detected by ELISA. * means *p <* 0.05 compared with sh-NC group; # means *p <* 0.05 compared with sh-GALNT1 group; There were 6 mice in each group

To further investigate the role of the GALNT1/RPRD1A axis in GC immune escape and peritoneal metastasis, highly metastatic MKN74 and GC9811-P cells with the indicated genetic modifications were subcutaneously inoculated into HIS mice. Tumor growth was monitored weekly. After 6 weeks, tumors were excised, photographed, and weighed. Similar trends were observed in both MKN74- and GC9811-P-derived tumors. Compared with the sh-NC group, tumors in the sh-GALNT1 group grew more slowly and had significantly lower tumor weights. In contrast, RPRD1A overexpression in GALNT1-silenced cells partially restored tumor growth and tumor weight (Fig. 4A-B, Figure S8A-B).

We next examined the expression of GALNT1 and RPRD1A in tumor tissues. Compared with the control group, the expression levels of both GALNT1 and RPRD1A were markedly reduced in tumors derived from GALNT1-silenced cells. Notably, RPRD1A overexpression partially rescued RPRD1A expression in the sh-GALNT1 + oe-RPRD1A group (Fig. 4C, Figure S8C).

To assess the effect of the GALNT1/RPRD1A axis on peritoneal dissemination, MKN74 and GC9811-P cells were injected intraperitoneally to establish peritoneal metastasis models in HIS mice. Four weeks later, mesenteric metastatic lesions were harvested and quantified by weight. Relative to the sh-NC group, GALNT1 knockdown significantly reduced both the number and weight of mesenteric metastatic tumors. These inhibitory effects were partially reversed by RPRD1A overexpression (Fig. 4D, Figure S8D).

We further evaluated immune cell infiltration in metastatic lesions by immunohistochemistry. GALNT1 knockdown markedly increased intratumoral infiltration of CD4^+^ and CD8^+^ T cells, whereas RPRD1A overexpression reduced CD4⁺ and CD8⁺ T-cell infiltration compared with the sh-GALNT1 group (Fig. 4E, Figure S8E). Consistently, ELISA analysis of ascites showed that GALNT1 silencing significantly increased the levels of IFN-γ, TNF-α, and Gzmb compared with the control group, whereas these increases were attenuated in the sh-GALNT1 + oe-RPRD1A group (Fig. 4F, Figure S8F).

Overall, these results indicate that the GALNT1/RPRD1A axis promotes GC progression, peritoneal metastasis, and immune evasion in vivo.

### GC9811-P-secreted EVs transfer GALNT1 to Promote migration of GC cells and suppress activation of CD8^+^ T Cells

**Fig. 5:**
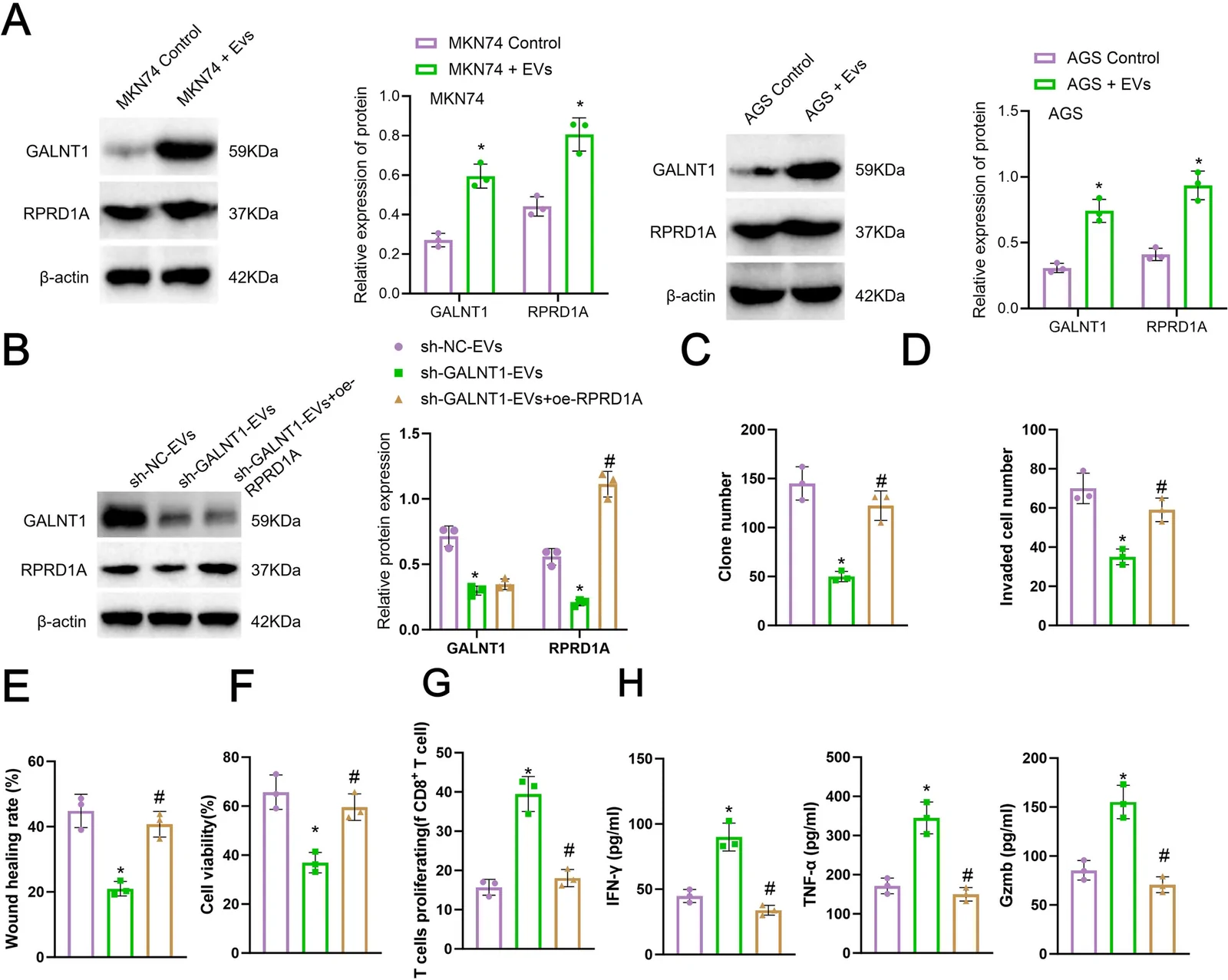
Effects of GC9811-P-EVs carrying GALNT1 to GC cells on their biological functions. Note: **A** Western blot analysis of GALNT1 and RPRD1A expression in MKN74 and AGS cells co-cultured with EVs; **B** Western blot analysis of GALNT1 and RPRD1A expression in AGS cells with or without RPRD1A overexpression after co-culture with GALNT1-deficient EVs; **C** Colony formation assay; **D** Transwell invasion assay (scale bar = 50 μm); **E** Wound-healing assay; **F** tumor-cell viability after co-culture with CD8 + T cells; **G** CD8 + T-cell proliferation after co-culture; **H** ELISA detection of IFN-γ, TNF-α, and Gzmb in co-culture supernatants. *p < 0.05 compared with the sh-NC-EVs group; #*p <* 0.05 compared with the sh-GALNT1-EVs group. Cell experiments were repeated three independent times

To further determine whether highly metastatic GC9811-P cells promote GC progression and immune evasion through EV-mediated transfer of GALNT1, we co-cultured GC9811-P-derived EVs with MKN74 and AGS cells. After co-culture, both GALNT1 and RPRD1A expression levels were increased in recipient MKN74 and AGS cells (Fig. 5A), indicating that GC9811-P-EVs carry GALNT1 and can be internalized by recipient GC cells to enhance RPRD1A expression.

To clarify how GALNT1 is incorporated into EVs, we first examined its intracellular localization. Immunofluorescence analysis showed that GALNT1 was predominantly localized to the Golgi apparatus in GC9811-P cells and strongly colocalized with the Golgi marker GM130 (Figure S9A). We then isolated the multivesicular body (MVB) fraction and found by WB that GALNT1 was enriched in the MVB fraction together with the exosomal marker CD63, whereas the Golgi marker GM130 was barely detectable (Figure S9B). These findings indicate that GALNT1 is selectively recruited into MVBs before EV secretion. Consistently, treatment with the exosome secretion inhibitor GW4869 markedly reduced the levels of both CD63 and GALNT1 in EVs compared with the DMSO control (Figure S9C). Together, these results indicate that GALNT1 is not secreted through the conventional secretory pathway, but is selectively packaged into EVs through the endosome–MVB–exosome biogenesis pathway.

To investigate whether GC9811-P-EVs can transfer GALNT1 to MKN74 and AGS cells, GALNT1 was knocked down in GC9811-P cells before EV isolation. The resulting EVs were incubated with MKN74 and AGS cells to detect GALNT1 and RPRD1A expression in each group. After co-incubation with GALNT1-deficient GC9811-P-EVs, GALNT1 and RPRD1A expression was reduced in MKN74 and AGS cells, whereas RPRD1A overexpression in recipient cells restored RPRD1A expression (Fig. 5B, Figure S10A).

Functional assays further showed that treatment with GALNT1-deficient GC9811-P-EVs significantly reduced the proliferative, invasive, and migratory capacities of both MKN74 and AGS cells, as demonstrated by colony formation, Transwell, and wound-healing assays (Fig. 5C–E and Figure S10B–D). Re-expression of RPRD1A in recipient cells partially rescued these malignant phenotypes, indicating that the prometastatic effects of GC9811-P-EVs are mediated, at least in part, through the GALNT1/RPRD1A axis.

We next examined whether this EV-mediated signaling pathway also regulates antitumor immunity. CD8^+^ T cells were co-cultured with AGS and MKN74 cells treated with GC9811-P-EVs or GALNT1-deficient EVs. Compared with the sh-NC-EVs group, sh-GALNT1-EVs significantly enhanced T-cell-mediated tumor cell killing (Fig. 5F, Figure S10E). Flow cytometry and ELISA further showed that sh-GALNT1-EV treatment increased CD8^+^ T-cell proliferation and elevated the levels of IFN-γ, TNF-α, and Gzmb (Fig. 5G-H, Figure S10F-G). In contrast, overexpression of RPRD1A in recipient MKN74 and AGS cells largely reversed the enhanced T-cell killing and CD8⁺ T-cell activation induced by sh-GALNT1-EVs (Fig. 5F-H, Figure S10E-G).

Collectively, these results demonstrate that GC9811-P-derived EVs transfer GALNT1 to recipient GC cells, thereby upregulating RPRD1A, enhancing metastatic potential, and suppressing CD8^+^ T-cell activation.

### GC9811-P-EVs carrying GALNT1 promote immune evasion and peritoneal metastasis of GC cells

**Fig. 6:**
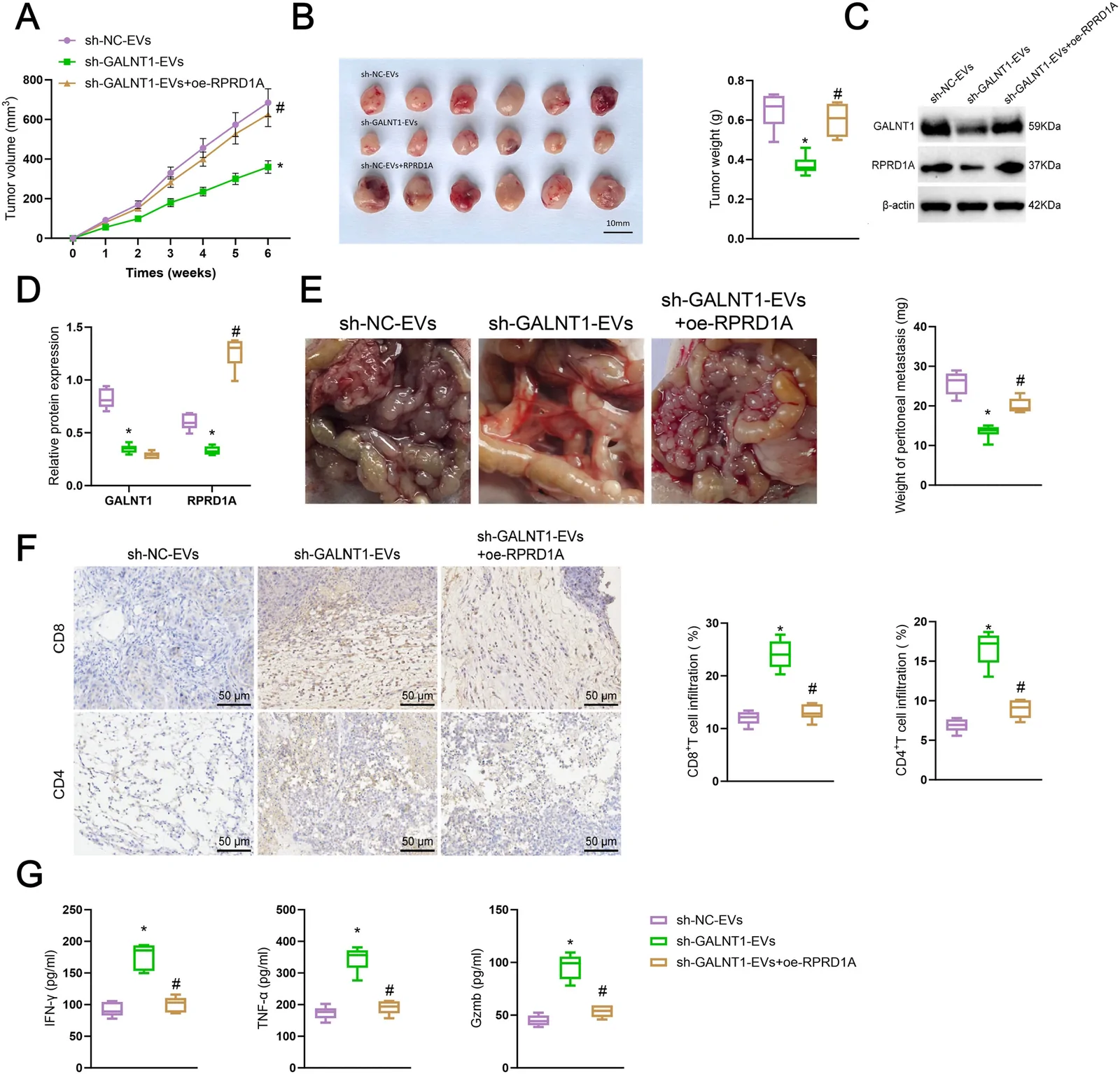
Effect of GC9811-P-EVs on immune escape and peritoneal metastasis of MKN74 cells. Note: **A** Tumor growth curves; **B** tumor weight statistics; **C**-**D** Western blot analysis of GALNT1 and RPRD1A expression in tumor tissues; **E** representative mesenteric metastatic lesions and weight quantification; **F** IHC analysis of CD8 + and CD4 + T-cell infiltration in mesenteric tumors; **G** ELISA detection of IFN-γ, TNF-α, and Gzmb in ascites. *p < 0.05 compared with the sh-NC-EVs group; #*p <* 0.05 compared with the sh-GALNT1-EVs group. Six mice per group

To further determine whether GC9811-P-derived EVs promote GC progression in vivo through GALNT1, MKN74 cells were subcutaneously inoculated into HIS mice, followed by weekly tail-vein injection of the indicated EVs (Fig. 6A). Tumor growth was monitored over time, and tumors were harvested and weighed after 6 weeks (Fig. 6B). Compared with the sh-NC-EVs group, mice treated with sh-GALNT1-EVs showed slower tumor growth and significantly reduced tumor weight. In contrast, overexpression of RPRD1A in recipient tumor cells partially restored the tumor-promoting effect of GC9811-P-EVs, as reflected by increased tumor growth and tumor weight relative to the sh-GALNT1-EVs group.

WB analysis of tumor tissues further showed that treatment with sh-GALNT1-EVs reduced RPRD1A expression compared with sh-NC-EVs, whereas RPRD1A overexpression restored its expression in the rescue group (Fig. 6C, D). These results support the notion that EV-derived GALNT1 promotes tumor growth through upregulation of RPRD1A.

To investigate the effect of GC9811-P-EVs on peritoneal dissemination, a peritoneal metastasis model was established by intraperitoneal injection of MKN74 cells into HIS mice. At the 4-week endpoint, mesenteric metastatic nodules were excised and subjected to weight measurement. Compared with the sh-NC-EVs group, treatment with sh-GALNT1-EVs significantly reduced the number and weight of mesenteric metastatic tumors. These inhibitory effects were partially reversed in the RPRD1A rescue group (Fig. 6E).

We next assessed immune cell infiltration in mesenteric metastatic lesions by immunohistochemistry. Compared with the sh-NC-EVs group, the sh-GALNT1-EVs group showed increased infiltration of both CD4^+^ and CD8^+^ T cells. In contrast, RPRD1A overexpression reduced CD4^+^ and CD8^+^ T-cell infiltration relative to the sh-GALNT1-EVs group (Fig. 6F). Consistently, ELISA analysis of ascites showed that GALNT1-deficient EVs significantly increased the levels of IFN-γ, TNF-α, and Gzmb, whereas these effects were attenuated after RPRD1A restoration (Fig. 6G).

GALNT1-deficient GC9811-P-EVs downregulated RPRD1A expression and attenuated immune escape and peritoneal metastasis, whereas RPRD1A overexpression partially rescued these effects.

### Preparation and characterization of LNPs@GALNT1 siRNA

**Fig. 7:**
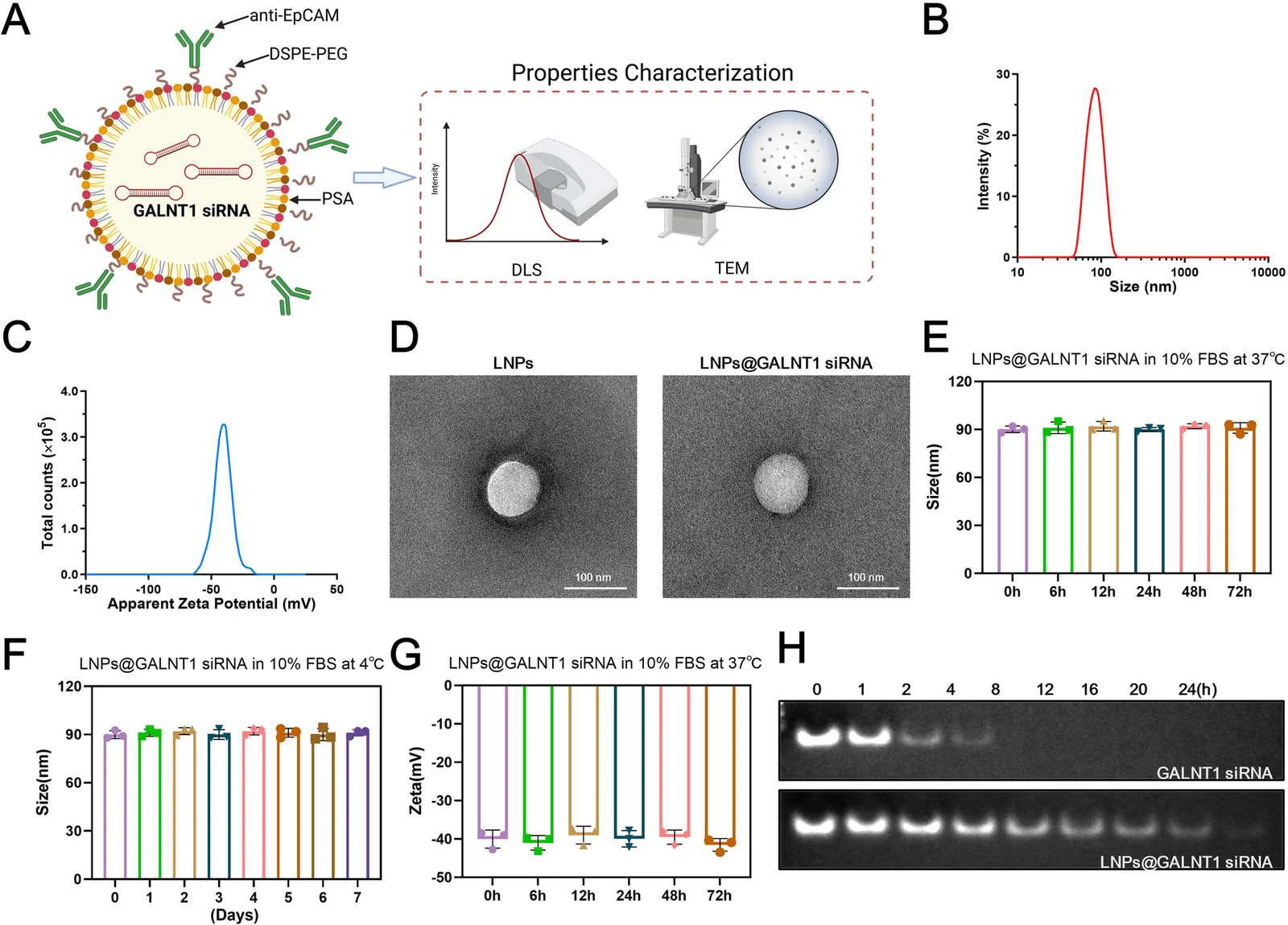
Preparation and physicochemical characterization of LNPs@GALNT1 siRNA. Note: **A** Schematic diagram of LNPs@GALNT1 siRNA construction; **B** Particle size distribution of LNPs@GALNT1 siRNA measured by DLS; **C** Zeta potential analysis of the surface charge of LNPs@GALNT1 siRNA; **D** Morphological structure of LNPs@GALNT1 siRNA observed by TEM (bar = 100 nm); **E** Particle size variation of LNPs@GALNT1 siRNA during 72 h incubation in PBS at 37 °C with 10% FBS; **F** Particle size stability of LNPs@GALNT1 siRNA during 7-day storage at 4 °C; **G** Zeta potential variation of LNPs@GALNT1 siRNA after 72 h incubation at 37 °C; **H** Stability comparison between LNPs@GALNT1 siRNA and naked siRNA after 24 h incubation in FBS. The assay was repeated three independent times

Given the poor stability, limited membrane permeability, and low in vivo targeting efficiency of free siRNA, we constructed an LNP-based delivery system encapsulating GALNT1-targeting siRNA to improve siRNA stability and cellular uptake, thereby enabling efficient GALNT1 silencing in GC cells. To further evaluate the therapeutic potential of GALNT1 targeting in GC immune escape and peritoneal metastasis, LNPs@GALNT1 siRNA were prepared and systematically characterized (Fig. 7A).

DLS analysis revealed that the nanoparticles had an average diameter of 85.2 ± 2.1 nm with good uniformity, and a zeta potential of − 40.87 ± 1.73 mV, indicating strong colloidal stability (Fig. 7B-C). TEM further showed that the nanoparticles displayed a uniform near-spherical morphology with clear boundaries and a concentrated size distribution (Fig. 7D). Stability assays demonstrated that, after incubation in PBS containing 10% FBS at 37 °C for up to 72 h, LNPs@GALNT1 siRNA showed minimal changes in particle size (Fig. 7E). Particle size also remained stable during storage at 4 °C for 7 days (Fig. 7F), and no significant fluctuation in zeta potential was observed after 72 h at 37 °C (Fig. 7G), indicating favorable physiological and storage stability.

We next examined the siRNA-protective capacity of the formulation. Free GALNT1 siRNA was almost completely degraded within 4 h in PBS containing 10% FBS, whereas siRNA encapsulated in LNPs remained detectable for up to 20 h (Fig. 7H), indicating that LNP encapsulation markedly delayed siRNA degradation in vitro.

To further assess in vivo biosafety, histopathological, hematological, and serum biochemical analyses were performed in C57BL/6 mice treated with LNPs@GALNT1 siRNA (Figure S11A). H&E staining showed that the heart, liver, spleen, lungs, and kidneys of LNPs@GALNT1 siRNA-treated mice retained intact tissue architecture, with no evidence of inflammatory infiltration, necrosis, or fibrosis (Figure S11B). Hematological analysis revealed no significant differences in RBC, WBC, HGB, or PLT counts between the LNP-treated and control groups, indicating that the material did not adversely affect the hematopoietic system (Figure S11C). Furthermore, serum biochemical analyses demonstrated that ALT, AST, BUN, and CRE levels remained within normal ranges after LNP treatment, suggesting no apparent impairment of hepatic or renal function (Figure S11D). Collectively, these findings indicated that LNPs@GALNT1 siRNA exhibited favorable biocompatibility and safety in vivo.

### In vivo distribution of LNPs@GALNT1 siRNA

**Fig. 8:**
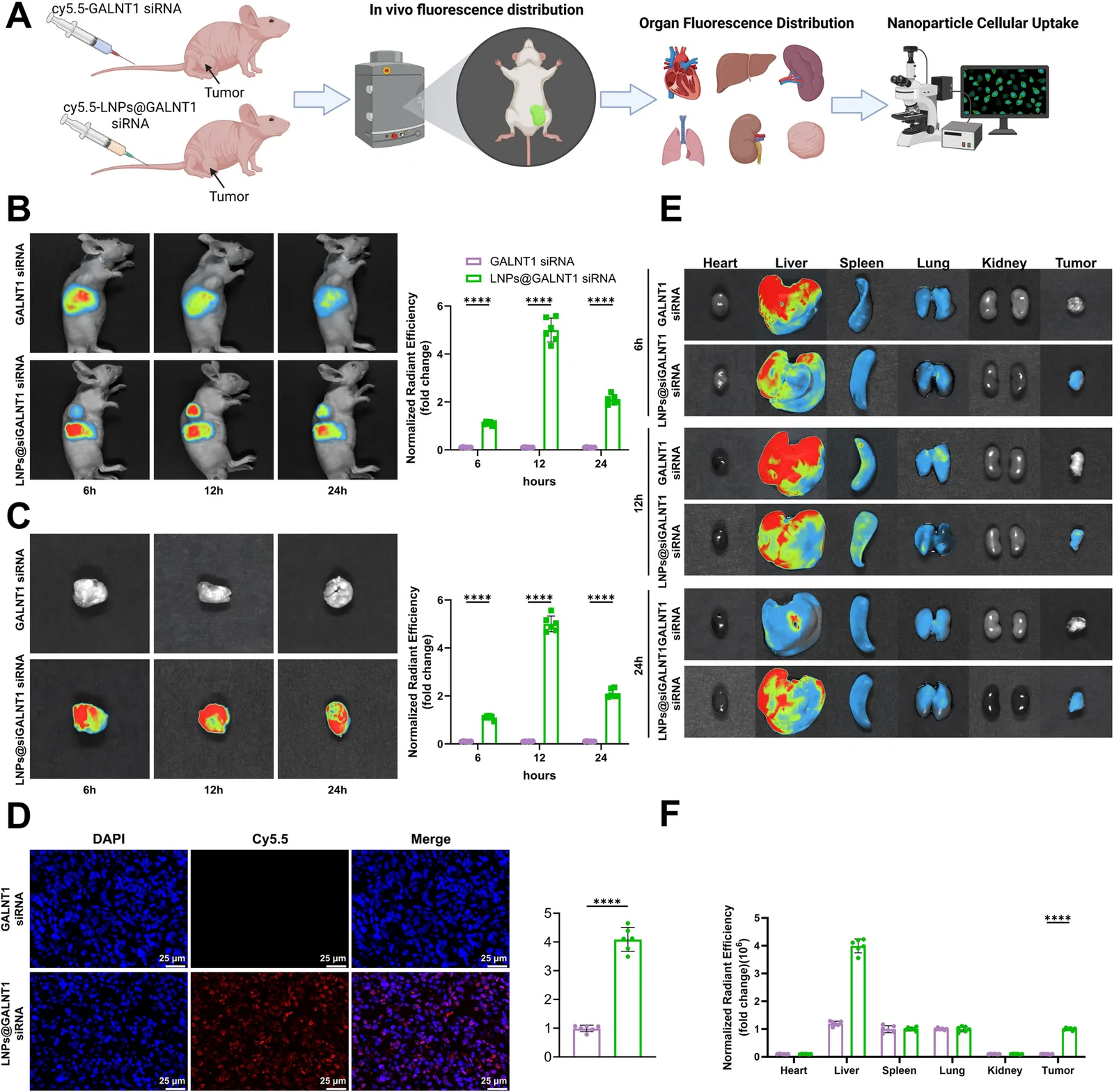
In vivo distribution profile and tumor accumulation of LNPs@GALNT1 siRNA. Note: **A** Fluorescence distribution of Cy5.5-labeled free GALNT1 siRNA and LNPs@GALNT1 siRNA in subcutaneous GC xenograft mice monitored by the IVIS imaging system; **B** Fluorescence accumulation of Cy5.5-labeled LNPs@GALNT1 siRNA in the tumor region at different time points (6 h, 12 h, 24 h); **C** Ex vivo fluorescence imaging of tumor tissues and quantitative analysis of fluorescence intensity; **D** Distribution of Cy5.5-siRNA within tumor tissues detected by fluorescence microscopy of frozen sections (red: Cy5.5-siRNA, blue: DAPI); **E** Ex vivo fluorescence imaging of major organs (heart, liver, spleen, lungs, kidneys) and tumor tissues to evaluate biodistribution; **F** Quantitative analysis of fluorescence intensity in different organs at 24 h post-injection. **p < 0.01, ****p < 0.0001

To evaluate the biodistribution and tumor accumulation profile of LNPs@GALNT1 siRNA, a subcutaneous GC xenograft mouse model was established, followed by tail vein injection of Cy5.5-labeled free GALNT1 siRNA or LNPs-loaded GALNT1 siRNA (Fig. 8A). In vivo fluorescence imaging showed that free Cy5.5-labeled GALNT1 siRNA was rapidly cleared after injection and exhibited minimal accumulation in the tumor region. In contrast, Cy5.5-labeled LNPs@GALNT1 siRNA showed sustained enrichment at the tumor site at 6, 12, and 24 h after injection, indicating improved in vivo stability and tumor accumulation (Fig. 8B). Further fluorescence imaging and quantitative analysis of excised tumor tissues confirmed that the LNP delivery system significantly enhanced siRNA accumulation within tumors (Fig. 8C).

Frozen tumor sections collected 12 h after injection were then analyzed by fluorescence microscopy. Cy5.5-labeled siRNA was broadly distributed throughout tumor tissues, and the fluorescence intensity in the LNPs@GALNT1 siRNA group was markedly higher than that in the free siRNA group (Fig. 8D), further supporting the superior delivery efficiency and intratumoral penetration of the LNP formulation.

To determine organ distribution, the heart, liver, spleen, lungs, kidneys, and tumors were collected at 6, 12, and 24 h post-injection for ex vivo fluorescence imaging. Tumors from the LNPs@GALNT1 siRNA group exhibited markedly stronger fluorescence signals than those from the free siRNA group. Higher fluorescence intensity was also detected in the liver and spleen, consistent with uptake by the reticuloendothelial system (Fig. 8E-F).

Collectively, these results demonstrate that LNPs@GALNT1 siRNA possess favorable in vivo stability and effectively accumulate in tumor tissues, supporting their potential for subsequent therapeutic application.

### LNPs@GALNT1 siRNA Suppressed Tumor Growth and Enhanced Antitumor Immune Responses

**Fig. 9:**
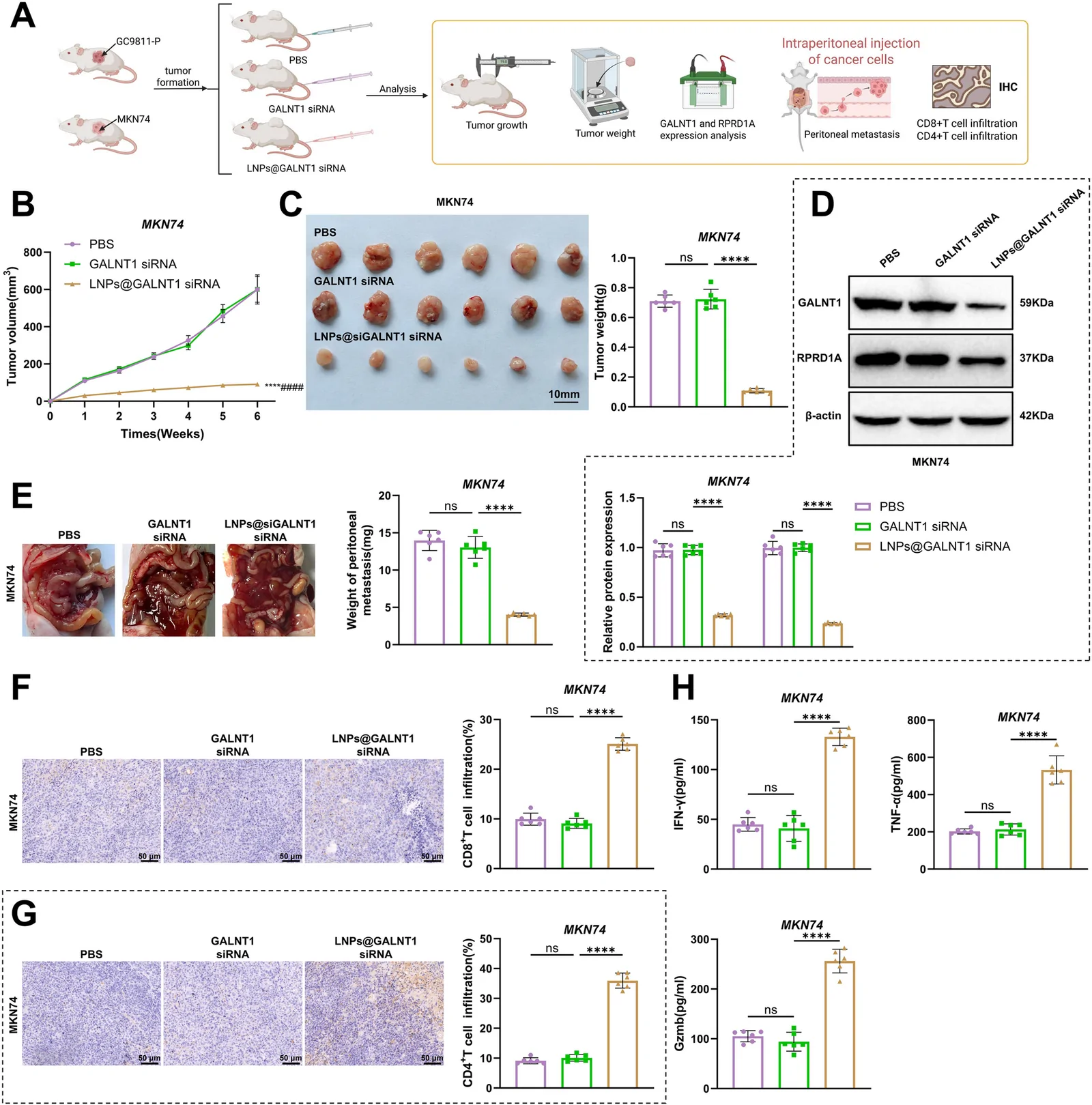
In vivo antitumor and immunomodulatory effects of LNPs@GALNT1 siRNA in HIS mouse models established with MKN74 cells. Note: **A** Experimental design and workflow for evaluating the in vivo antitumor and immunomodulatory effects of LNPs@GALNT1 siRNA in HIS mouse models established with MKN74 cells; **B** Tumor volume measurements of subcutaneous MKN74 xenografts using calipers and tumor growth curves (*p < 0.05, ***p < 0.001 vs. PBS; #*p <* 0.05, ##*p <* 0.01 vs. GALNT1 siRNA); **C** Representative tumor images from each group and tumor weights measured with an electronic balance; **D** Western blot analysis of GALNT1 and RPRD1A protein expression levels in tumors derived from subcutaneous MKN74 xenografts; **E** Representative images of mesenteric tumor nodules from each group and nodule weights measured with an electronic balance; **F** IHC analysis of CD8⁺ T cell infiltration in mesenteric tumor nodules from mice intraperitoneally injected with MKN74 cells (bar = 50 μm); **G** IHC analysis of CD4⁺ T cell infiltration in mesenteric tumor nodules from mice intraperitoneally injected with MKN74 cells (bar = 50 μm); **H** ELISA analysis of IFN-γ, TNF-α, and Gzmb levels in ascites from mice intraperitoneally injected with MKN74 cells. ****p < 0.0001. Six mice per group

To evaluate the antitumor efficacy of LNPs@GALNT1 siRNA in vivo and its effects on the immune microenvironment, therapeutic studies were performed in HIS mouse models. HIS mice were subcutaneously inoculated with MKN74 or GC9811-P cells and randomly assigned to the PBS, GALNT1 siRNA, or LNPs@GALNT1 siRNA groups (Fig. 9A). Tumor growth monitoring showed no significant difference between the GALNT1 siRNA and PBS groups, whereas tumor growth was markedly suppressed in the LNPs@GALNT1 siRNA group (Fig. 9B & Figure S12A). Consistently, tumor weights at the endpoint were comparable between the GALNT1 siRNA and PBS groups but were significantly reduced after LNPs@GALNT1 siRNA treatment (Fig. 9C & Figure S12B). WB analysis of tumor tissues further showed that GALNT1 and RPRD1A expression remained largely unchanged in the GALNT1 siRNA group, but both were markedly decreased in the LNPs@GALNT1 siRNA group (Fig. 9D & Figure S12C).

We next assessed the therapeutic effects of LNPs@GALNT1 siRNA in peritoneal metastasis models established by intraperitoneal injection of MKN74 or GC9811-P cells. The number and weight of mesenteric metastatic nodules were not significantly altered in the GALNT1 siRNA group compared with the PBS group, but were markedly reduced in the LNPs@GALNT1 siRNA group (Fig. 9E & Figure S12D). Immunohistochemical analysis of mesenteric tumor nodules showed no significant difference in T cell infiltration between the GALNT1 siRNA and PBS groups, while CD8^+^ and CD4^+^ T cell infiltration was significantly increased in the LNPs@GALNT1 siRNA group (Fig. 9F-G & Figure S12E-F). Consistently, ELISA analysis of ascites showed that IFN-γ, TNF-α, and Gzmb levels were not significantly changed in the GALNT1 siRNA group but were markedly elevated in the LNPs@GALNT1 siRNA group (Fig. 9H & Figure S12G). Consistent results were obtained in models established with both MKN74 and GC9811-P cells.

Collectively, these results indicate that free GALNT1 siRNA alone has limited antitumor efficacy in vivo, whereas LNP-mediated delivery effectively suppresses GALNT1/RPRD1A signaling, enhances T-cell infiltration and activation, and inhibits GC growth and peritoneal metastasis.

## Discussion

Peritoneal metastasis is a major determinant of poor prognosis in GC, and substantial effort has been devoted to elucidating its molecular basis and metastatic routes. EVs are key mediators of intercellular communication and have been widely implicated in the regulation of tumor aggressiveness and metastatic spread across multiple cancer types [15, 62]. This study demonstrates that GALNT1-mediated glycosylation of RPRD1A in EVs derived from highly metastatic GC cells markedly promotes peritoneal metastasis. These findings deepen current understanding of the molecular basis of GC peritoneal dissemination and highlight a potential therapeutic target. Previous studies have largely focused on the transfer of miRNAs, lncRNAs, or proteins by EVs during cancer progression [63–67], whereas the role of EV-associated glycosylation events in tumor metastasis has received far less attention. Therefore, this study provides a new perspective on the contribution of EV-mediated glycosylation to metastatic progression.

GALNT1 is a glycosyltransferase, and its expression in various tumors positively correlates with the malignant degree of tumors [28, 29]. GALNT1 regulates the biological behavior of tumor cells by affecting protein abundance and function through glycosylation [27, 61]. In the present study, we found that GALNT1 may promote immune escape and peritoneal metastasis of GC through glycosylation of RPRD1A. This mechanism differs, at least in part, from those reported in other tumor types, in which GALNT1 has mainly been implicated in altering cell-cell interactions through the glycosylation of cell-surface molecules. Our findings suggest that GALNT1 can be transferred through EVs and remodel the TME in recipient cells. This observation expands the current understanding of EV-mediated tumor regulation and highlights a previously underappreciated link between EV cargo glycosylation and metastatic progression.

This study further expands the understanding of EV function in GC and underscores their important role in shaping the TME. We found that EVs derived from highly metastatic GC cells markedly enhanced the migratory and invasive capacities of recipient GC cells in vitro. These findings suggest that tumor cells can indirectly remodel the TME by altering the biological properties of EVs, thereby facilitating tumor dissemination and metastatic progression.

Regulation of the immune microenvironment is a central issue in tumor biology, particularly because immune escape critically limits the efficacy of anticancer therapy. In the present study, we found that GC cells transmit specific molecular signals through EV secretion, especially via GALNT1-mediated glycosylation of RPRD1A, thereby modulating immune cell function and promoting immune evasion. This mechanism provides a new perspective on how tumors sustain survival and metastatic spread within the host. Previous studies have mainly focused on the roles of intracellular signaling pathways and cytokines in immune escape [68–70], whereas our findings highlight the distinct contribution of EV-mediated signaling to this process. These results may provide a theoretical basis for the development of novel immunotherapeutic strategies against GC.

Our study suggests that highly metastatic GC cells may establish a multilayered immunosuppressive network through the secretion of EVs enriched in GALNT1. The underlying mechanism appears to involve both the reprogramming of tumor cell-intrinsic properties to enhance resistance to immune attack and the suppression of antitumor T-cell responses, thereby generating a coordinated immune-evasive effect. By increasing RPRD1A protein abundance, this pathway markedly enhanced tumor cell migration, invasion, and peritoneal colonization, which may facilitate dissemination to sites with relatively limited immune surveillance and thereby contribute to immune escape. Together with the broad impairment of T-cell function observed in our study, these findings further imply that this regulatory network may also exert direct or indirect inhibitory effects on T cells.

After co-culture with tumor cells regulated by this axis, CD8 + T cells exhibited not only reduced cytotoxic activity, but also markedly impaired proliferation and activation. These findings suggest that tumor cells regulated by the GALNT1/RPRD1A axis, or the EVs they release, may indirectly create an immunosuppressive environment that limits CD8 + T-cell proliferation and effector cytokine production. Whether GALNT1 packaged within EVs can be directly taken up by T cells and alter the glycosylation status of key proteins involved in T-cell receptor signaling remains an important question for future investigation.

Taken together, the GALNT1/RPRD1A axis may exert synergistic effects through a dual mechanism. At the level of tumor cells, it may reinforce immune resistance by enhancing metastatic capacity and shaping an immunosuppressive tumor microenvironment. At the level of effector T cells, it may attenuate antitumor activity through direct or indirect inhibitory interactions. Such a ”double-hit” model may jointly drive immune escape and peritoneal metastasis. Future studies using blocking co-culture experiments and analyses of T-cell signaling pathways will be needed to define the specific immune mediators involved. Nevertheless, targeting the upstream regulator GALNT1 was sufficient to reverse multiple downstream phenotypes, underscoring the central role of this axis in shaping the immunosuppressive microenvironment of GC.

## Conclusion

**Fig. 10:**
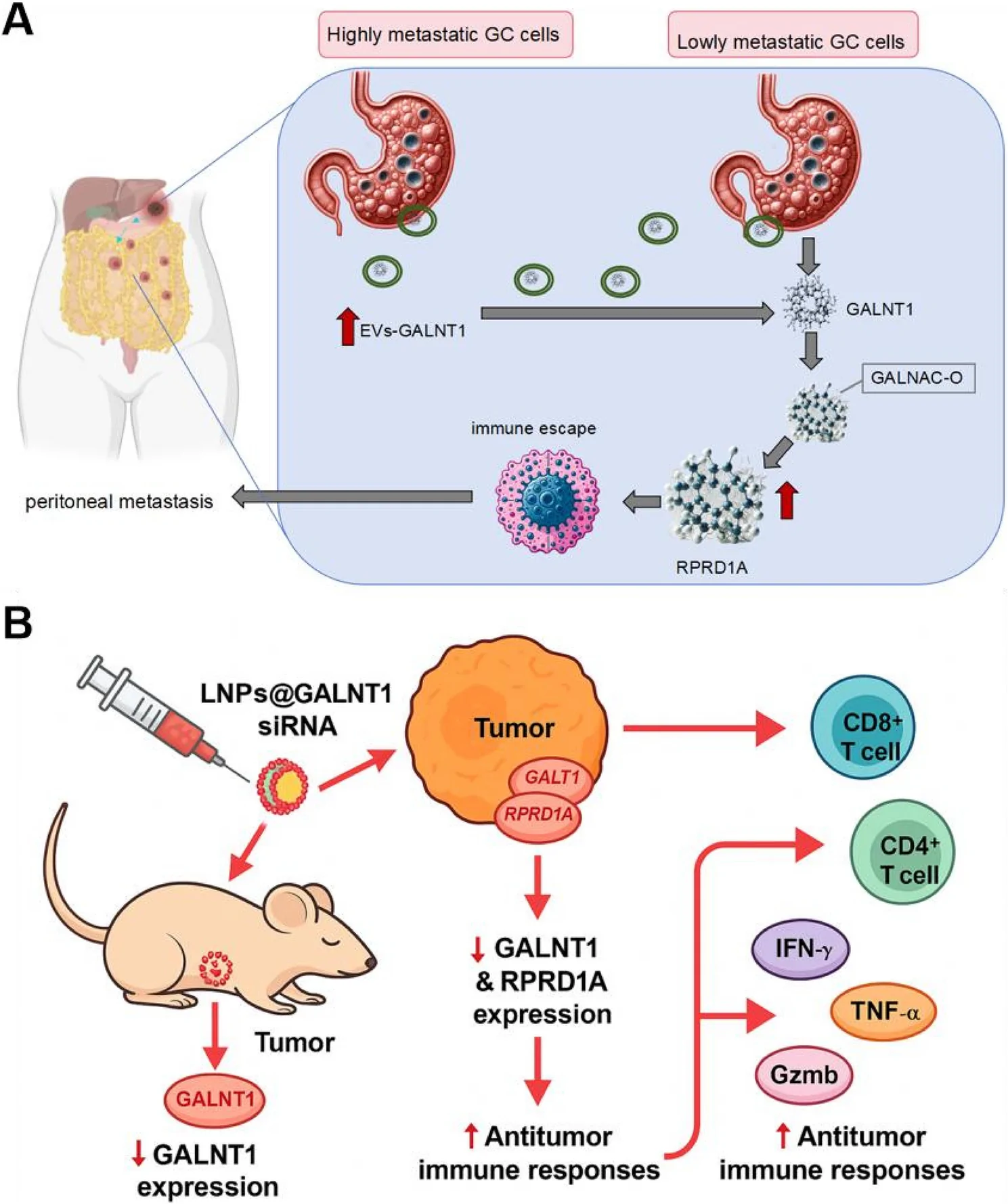
Mechanisms of immune escape mediated by EVs from highly metastatic GC cells and therapeutic effects of LNPs@GALNT1 siRNA. Note: **A** EVs secreted by highly metastatic GC cells were enriched in GALNT1 and transferred to low-metastatic GC cells, leading to increased O-GalNAc modification and upregulation of RPRD1A protein expression, thereby inducing immune escape and promoting peritoneal metastasis. **B** Systemic administration of LNPs@GALNT1 siRNA enabled delivery into tumor tissues, inhibited GALNT1 expression, and reduced RPRD1A expression, while enhancing CD8⁺ and CD4⁺ T cell infiltration and promoting IFN-γ, TNF-α, and Gzmb secretion, ultimately augmenting antitumor immune responses and suppressing GC growth and peritoneal metastasis

In summary, our study shows that EVs derived from highly metastatic GC cells transfer GALNT1 to recipient tumor cells, thereby increasing RPRD1A protein expression through GALNT1-dependent O-GalNAc modification and promoting immune escape and peritoneal metastasis (Fig. 10A). Targeting this pathway with LNPs@GALNT1 siRNA effectively enhanced antitumor immunity and suppressed GC growth and peritoneal dissemination (Fig. 10B). These findings identify the GALNT1/RPRD1A axis as a previously unrecognized mechanism underlying GC metastasis and support its potential as a therapeutic target. Despite these promising results, further investigation will be required to address GC heterogeneity, dissect the complex tumor–immune interactions involved, and determine the translational potential of this strategy in clinical practice.

## Supplementary Information

Supplementary Material 1: Figure S1. Detection of GC cell invasion and migration and EV identification. Note: (A) The migration ability of AGS, SNU-520, FU97, SNU-719, MKN74 and GC9811-P cells was detected by cell scratch assay, * indicating *p* < 0.05 for comparison between the two groups; (B) The invasion ability of AGS, SNU-520, FU97, SNU-719, MKN74 and GC9811-P cells was detected by Transwell assay (scale bar = 50 μm), * indicating *p* < 0.05 for comparison between the two groups; (C) The morphology of GC9811-P-EVs was observed under TEM (scale bar = 100 nm). (D) GC9811-P-EVs particle diameter detected by DLS; (E) Western blot analysis of CD63, HSP70 and Calnexin expression in GC9811-P-EVs; (F) After labeling GC9811-P-EVs with PKH26(red) and co-culture with MKN74 and AGS cells for 24 h, the internalized cell experiment on EVs was observed to be repeated at least three times.

Supplementary Material 2: Figure S2. Effects of GC9811-P-EVs on AGS cell metastasis and CD8+T cell function in vitro. Note: (A) Clonal formation assay detected cell proliferation in each group. (B) The invasion ability of cells in each group was detected by Transwell assay (scale bar = 50 μm). (C) The metastasis ability of cells in each group was detected by scratch test. (D) Proliferation capacity of tumor cells after co-culture with CD8+T cells; (E) Proliferation ability of CD8+T cells after co-culture; (F) The expression of immunorelated factors IFN-γ, TNF-α and Gzmb in the supernatant after ELISA detected co-culture. *p < 0.05 versus the PBS group; #*p* < 0.05 versus the GC9811-P-EVs group. The cell experiment was repeated three times.

Supplementary Material 3: Figure S3. Establishment procedure and timeline of the HIS mouse model. Note: (A) HIS mouse modeling process and timeline; (B) The proportion of hCD45+ cells in the peripheral blood of mice was detected by flow cytometry (NSG mouse n = 6, HIS mouse n = 6).

Supplementary Material 4: Figure S4. Bioinformatics screening of key targets associated with GC EVs. Note: (A) 20 GalNAc transferases (GALNTs) were differentially expressed in the volcano map of STAD samples in TCGA database; (B) Differential expression GALNTs heat map display, Normal, n = 37, Tumor, n = 375; (C) Demonstrate the expression of 6 highly expressed GALNTs in GCEVs based on public database; (D) Detection of GALNT1 protein content in EVs; (E) Sequencing of genes related to GALNT1; (F) The interaction map of 30 GALNT1 genes with significant correlation with GALNT1 protein; (G) Kaplan-Meier survival curves of patients with high expression of GALNT1 and RPRD1A (n = 68); (H) Western blot analysis and semi-quantitative analysis of GALNT1 and RPRD1A in GES-1, AGS, SNU-520, FU97, SNU-719, MKN74 and GC9811-P cells. *p < 0.05. The cell experiment was repeated at least three times.

Supplementary Material 5: Figure S5. Correlation analysis of GALNT1 and RPRD1A with immune cell infiltration in gastric cancer based on the CIBERSORT and TIMER databases. Note: (A-B) Correlation analysis of GALNT1 expression with immune cell infiltration in gastric cancer based on the CIBERSORT results and the TIMER database. (C-D) Correlation analysis of RPRD1A expression with immune cell infiltration in gastric cancer based on the CIBERSORT results and the TIMER database.

Supplementary Material 6: Figure S6. GALNT1 and RPRD1A expression in GC cells. Note: (A) Western blot analysis of GALNT1 knockdown efficiency in MKN74 and GC9811-P cells; (B) The expression of RPRD1A in AGS, MKN74 and GC9811-P cells was detected by RT-qPCR. (C) Western blot analysis of RPRD1A knockdown efficiency in AGS cells. (D-E) Western blot analysis of RPRD1A expression in AGS, MKN74 and GC9811-P cells. * means *p* < 0.05 compared with sh-NC/oe-NC group; # means *p* < 0.05 compared with sh-GALNT1 /oe-GALNT1 group. The cell experiment was repeated three times.

Supplementary Material 7: Figure S7. Repeated experiments of MKN74 cells (clonal formation, Transwell, scratch experiments). Note: (A) Clonal formation assay detected cell proliferation in each group. (B) The invasion ability of cells in each group was detected by Transwell assay (scale bar = 50 μm). (C) The metastasis ability of cells in each group was measured by scratch test. * means *p* < 0.05 compared with sh-NC group, and # means *p* < 0.05 compared with sh-GALNT1 group. The cell experiment was repeated three times.

Supplementary Material 8: Figure S8. Effects of GALNT1 regulation of RPRD1A expression on immune escape and metastasis ability of MKN74 cells. Note: (A) Tumor growth curve of mice in each group; (B) Representative pictures of tumor bodies and tumor weight statistics in each group; (C) The expression of GALNT1 and RPRD1A in the tumor of mice in each group was detected by Western blot. (D) The mesenteric tumors were observed and weighed in each group. (E) The infiltration of CD8+ and CD4+T cells in mesenteric tumors in each group was detected by immunohistochemical staining (scale bar = 50 μm). (F) ELISA detected the expression of immunorelated factors IFN-γ, TNF-α and Gzmb in ascites. * means *p* < 0.05 compared with sh-NC group; # means *p* < 0.05 compared with sh-GALNT1 group; There were 6 mice in each group.

Supplementary Material 9: Figure S9. Mechanism by which GALNT1 is packaged into extracellular vesicles. Note: (A) Immunofluorescence colocalization showing that GALNT1 (red) was highly colocalized with the Golgi marker GM130 (green) in GC9811-P cells. Nuclei were stained with DAPI (blue). Scale bar = 25 μm. (B) Western blot analysis of GALNT1, the exosomal marker CD63, the Golgi marker GM130, and the loading control β-actin in whole-cell lysates (WCL), multivesicular body (MVB) fractions, and purified EVs from GC9811-P cells. GALNT1 and CD63 were co-enriched in the MVB fraction and EVs. (C) GC9811-P cells were treated with DMSO (control) or the exosome secretion inhibitor GW4869 (10 µM) for 24 h, followed by EV isolation. Western blot analysis showed that GW4869 treatment markedly reduced the protein levels of GALNT1 and CD63 in EVs.

Supplementary Material 10: Figure S10. Repeated experiment of MKN74 cells. Note: (A) Western blot analysis of GALNT1 and RPRD1A expression in MKN74 cells co-cultured with GALNT1-deficient EVs, with or without RPRD1A overexpression; (B) Colony formation assay; (C) Transwell invasion assay (scale bar = 50 μm); (D) Wound-healing assay; (E) tumor-cell viability after co-culture with CD8 + T cells; (F) CD8 + T-cell proliferation after co-culture; (G) ELISA detection of IFN-γ, TNF-α, and Gzmb in co-culture supernatants. *p < 0.05 compared with the sh-NC-EVs group; #*p* < 0.05 compared with the sh-GALNT1-EVs group. Cell experiments were repeated three independent times.

Supplementary Material 11: Figure S11. In vivo biosafety assessment of LNPs@GALNT1 siRNA. Note: (A) Schematic diagram of the LNPs@GALNT1 siRNA treatment procedure; (B) H&E staining of major mouse organs (heart, liver, spleen, lungs, kidneys) to observe morphological changes (bar = 50 μm); (C) Hematological analysis of peripheral blood, including RBC, WBC, HGB, and PLT; (D) Serum biochemical analysis evaluating liver function (ALT, AST) and kidney function (BUN, CRE). Six mice per group.

Supplementary Material 12: Figure S12. In vivo antitumor and immunomodulatory effects of LNPs@GALNT1 siRNA in HIS mouse models established with GC9811-P cells. Note: (A) Tumor volume measurements of subcutaneous GC9811-P xenografts using calipers and tumor growth curves (*p < 0.05, ***p < 0.001 vs. PBS; #*p* < 0.05, ##*p* < 0.01 vs. GALNT1 siRNA); (B) Representative tumor images from each group and tumor weights measured with an electronic balance; (C) Western blot analysis of GALNT1 and RPRD1A protein expression levels in tumors derived from subcutaneous GC9811-*P* xenografts; (D) Representative images of mesenteric tumor nodules from each group and nodule weights measured with an electronic balance; (E) IHC analysis of CD8⁺ T cell infiltration in mesenteric tumor nodules from mice intraperitoneally injected with GC9811-*P* cells (bar = 50 μm); (F) IHC analysis of CD4⁺ T cell infiltration in mesenteric tumor nodules from mice intraperitoneally injected with GC9811-*P* cells (bar = 50 μm); (G) ELISA analysis of IFN-γ, TNF-α, and Gzmb levels in ascites from mice intraperitoneally injected with GC9811-P cells. **p < 0.01, ***p < 0.001, ****p < 0.0001. Six mice per group.

Supplementary Material 13.

## Data Availability

The data that support the findings of this study are available from the corresponding author upon reasonable request.

## Abbreviations

ALT: Alanine aminotransferase
AST: Aspartate aminotransferase
BCA: Bicinchoninic acid
BSA: Bovine serum albumin
BUN: Blood urea nitrogen
CCK-8: Cell Counting Kit-8
CFSE: Carboxyfluorescein succinimidyl ester
DAB: 3,3′-Diaminobenzidine
DAPI: 4′,6-Diamidino-2-phenylindole
DLS: Dynamic light scattering
DMEM: Dulbecco’s modified Eagle’s medium
ELISA: Enzyme-linked immunosorbent assay
EpCAM: Epithelial cell adhesion molecule
EVs: Extracellular vesicles
FBS: Fetal bovine serum
GALNT1: Polypeptide N-acetylgalactosaminyltransferase 1
GalNAc: N-acetylgalactosamine
GC: Gastric cancer
Gzmb: Granzyme B
H&E: Hematoxylin and eosin
HIS: Humanized immune system
IFN-γ: Interferon-γ
LNPs: Lipid nanoparticles
LNPs@GALNT1 siRNA: EpCAM-modified lipid nanoparticles loaded with GALNT1 siRNA
PBS: Phosphate-buffered saline
qPCR: Quantitative polymerase chain reaction
RIPA: Radioimmunoprecipitation assay
RPRD1A: Regulation of nuclear pre-mRNA domain containing 1 A
RT-qPCR: Reverse transcription quantitative polymerase chain reaction
TCGA: The Cancer Genome Atlas
TEM: Transmission electron microscopy
TME: Tumor microenvironment
TNF-α: Tumor necrosis factor-α
VVA: Vicia villosa agglutinin
WB: Western blot
