# Supplementary material for: Extracellular vesicle–mediated GALNT1/RPRD1A glycosylation axis drives immune escape and peritoneal metastasis in gastric cancer
Source: J Exp Clin Cancer Res. 2026 Jul 2;45:204. doi: 10.1186/s13046-026-03759-7 (PMC13602598; doi:10.1186/s13046-026-03759-7)
Supplement: Supplementary file 1 [file 13046_2026_3759_MOESM13_ESM.docx]

**Table S1. Clinicopathological features of GC patients.**

| **Features** | **Cases** |
| --- | --- |
| Median age (range) | 57 years (32-78) |
| Gender |  |
| Male | 40 (58.82%) |
| Female | 28 (41.18%) |
| Tumor size |  |
| < 5cm | 38 (55.88%) |
| ≥ 5cm | 30 (44.12%) |
| TNM stage |  |
| I | 15 (22.06%) |
| II | 24 (35.29%) |
| III | 26 (38.24%) |
| IV | 3 (4.41%) |
| Lymph node metastasis |  |
| N0 | 16 (23.53%) |
| N1 | 19 (27.94%) |
| N2 | 26 (38.24%) |
| N3 | 7 (10.29%) |
| Distant metastasis |  |
| M0 | 65 (95.59%) |
| M1 | 3 (4.41%) |

Note: GC, gastric cancer; TNM, tumor node metastasis.
**Table S2. shRNA sequences.**

| **shRNA** | **Sequence (5'-3')** |
| --- | --- |
| sh-NC | 5'-CCTAAGGTTAAGTCGCCCTCG-3' |
| sh-GALNT2-1 | 5'-CTCGACACTTTGGGACACTTT-3' |
| sh-GALNT2-2 | 5'-CCTTTCAAATGGTACCTTGAA-3' |
| sh-ENPP1-1 | 5'-CATGGGTTGAAGAATTGTTAA-3' |
| sh-ENPP1-2 | 5'-CTGCGAAAGTATGCTGAAGAA-3' |

Note: sh-, short hairpin RNA-; NC, negative control; GALNT2, polypeptide N-acetylgalactosaminyltransferase 2; ENPP1, ectonucleotide pyrophosphatase/phosphodiesterase 1.
**Table S3. Primer sequences for RT-qPCR.**

| **Gene** | **Sequence (5'-3')** |
| --- | --- |
| GALNT2 | Forward: 5'-GGCATTGCTGGGGTTAGGTA-3' |
|  | Reverse: 5'-CAGAGCCCTTTCCTCCCTTC-3' |
| ENPP1 | Forward: 5'-GGCATGGAACAAGGCAGTTG-3' |
|  | Reverse: 5'-TTGGTTCCCGGCAAGAAAGA-3' |
| GAPDH | Forward: 5'-AATGGGCAGCCGTTAGGAAA-3' |
|  | Reverse: 5'-GCGCCCAATACGACCAAATC-3' |

Note: RT-qPCR, reverse-transcriptase quantitative real-time polymerase chain reaction; GALNT2, polypeptide N-acetylgalactosaminyltransferase 2; ENPP1, ectonucleotide pyrophosphatase/phosphodiesterase 1; GAPDH, glyceraldehyde-3-phosphate dehydrogenase.
**Table S4. Manufacturer information of primary antibodies.**

| **Antibody** | **Cat.** | **Dilution ratio** | **Manufacturer** | **Country** |
| --- | --- | --- | --- | --- |
| Rabbit anti-GALNT2 | ab262868 | 1:1000 | Abcam | UK |
| Rabbit anti-ENPP1 | ab217368/ab223268 | 1:500 | Abcam | UK |
| Rabbit anti-p-PI3K | #4249 | 1:1000 | Abcam | UK |
| Mouse anti-PI3K | ab140307 | 1:2000 | Abcam | UK |
| Rabbit anti-p-AKT | ab38449 | 1:1000 | Abcam | UK |
| Rabbit anti-AKT | ab8805 | 1:1000 | Abcam | UK |
| Rabbit anti-p-mTOR | ab109268 | 1:1000 | Abcam | UK |
| Rabbit anti-mTOR | ab134903 | 1:10000 | Abcam | UK |
| Rat anti-GAPDH | ab8245 | 1:5000 | Abcam | UK |
| Rabbit anti-NA/K-ATPase | #3010 | 1:1000 | Abcam | UK |

Note: GALNT2, polypeptide N-acetylgalactosaminyltransferase 2; ENPP1, ectonucleotide pyrophosphatase/phosphodiesterase 1; p-, phosphorylated-; PI3K, phosphatidylinositol 3-kinase, putative; mTOR, mechanistic target of rapamycin kinase; GAPDH, glyceraldehyde-3-phosphate dehydrogenase.

**Table S5. Correlation of GALNT2 and ENPP1 expression with clinicopathologic features of GC patients.**

| **Features** | **GALNT2 expression** | | | **ENPP1 expression** | | |
| --- | --- | --- | --- | --- | --- | --- |
|  | **High (34)** | **Low (34)** | **p** | **High (34)** | **Low (34)** | **p** |
| Gender (Male:Female) | 18:16 | 22:12 | 0.324 | 21:13 | 19:15 | 0.622 |
| Tumor size (< 5cm:>= 5cm) | 31:3 | 7:27 | <0.001 | 10:24 | 28:6 | <0.001 |
| TNM stage (I + II:III + IV ) | 32:2 | 7:27 | <0.001 | 10:24 | 29:5 | <0.001 |
| Lymph node metastasis  (N0 + N1:N2 + N3) | 29:5 | 6:28 | <0.001 | 8:26 | 27:7 | <0.001 |

Note: GC, gastric cancer; TNM, tumor node metastasis; GALNT2, polypeptide N-acetylgalactosaminyltransferase 2; ENPP1, ectonucleotide pyrophosphatase/phosphodiesterase 1.
